# Improving paraffin precipitate inhibition using glycine and Palm-based Methyl Ester Sulfonate (MES) eco-friendly inhibitors

**DOI:** 10.1371/journal.pone.0313394

**Published:** 2025-01-28

**Authors:** Nazliah Binti Surpina, Mysara Eissa Mohyaldinn, Abdullah Abduljabbar, Mohammed Abdalla Ayoub

**Affiliations:** 1 Petroleum Engineering Department, Universiti Teknologi PETRONAS, Perak, Malaysia; 2 Center of Flow Assurance, Institute of Subsurface Resources, Universiti Teknologi PETRONAS, Perak, Malaysia; 3 Chemical & Petroleum Engineering Department, United Arab Emirates University, Al Ain, United Arab Emirates; Universidad San Francisco de Quito - Campus Cumbaya: Universidad San Francisco de Quito, ECUADOR

## Abstract

Oil fields located in cold environments and deep-sea locations often face challenges with paraffin wax buildup in pipelines during long-distance crude oil transportation. Various strategies have been employed to address this issue, with chemical methods being the most effective and economical. However, traditional chemical inhibitors present problems due to their high toxicity and low biodegradability, leading to increased operational costs and environmental concerns. This study focuses on developing an eco-friendly paraffin inhibitor system using three different concentrations of Glycine and Palm-based Methyl Ester Sulfonate (MES). Experiments were conducted on crude oil samples from the Dulang Oilfield. The experimental measurements include wax appearance temperature (WAT), pour point temperature (PPT), and rheological tests in the absence and presence of the proposed inhibitors. The results revealed that both Glycine and MES can effectively reduce WAT, viscosity, and yield point. Specifically, 10% Glycine was the best inhibitor, reducing WAT by 23.3%. However, MES (1%, 5%, and 10%) demonstrated greater overall effectiveness, with an average WAT reduction of 13.76% compared to Glycine’s 10.85%. MES also shows a better performance in reducing viscosity and yield stress. While PPT results were insignificant, MES is recommended as a flow improver rather than a pour point depressant. The successful development of these newly formulated chemical inhibitors promises an environmentally sustainable and economically efficient approach to maximizing oil production from mature fields while mitigating paraffin precipitation.

## 1. Introduction

Oil production in cold environments often requires long-distance crude oil transport, where wax buildup in pipelines can become a major issue if not properly addressed [1]. Wax accumulation affects production, transportation, and storage systems, causing flow assurance challenges like reduced pipe diameter, blockages, and increased viscosity, which requires higher pumping pressures [2, 3] (It can also lead to phase separation, altering the fluid’s composition and rheological properties, and reducing permeability near the wellbore [4–6]. Paraffin wax precipitates and deposits when straight-chain n-paraffin molecules in crude oil crystallize, cluster, and attach to cold pipeline walls. This occurs as the oil temperature drops to the wax appearance temperature (WAT), also called the cloud point, where waxy components crystallize into visible solid wax [1, 7]. Deposition continues as the temperature falls to the pour point (PP), where oil loses its flow ability, forming a gel-like structure with trapped oil and wax particles [8–10].

Various techniques have been developed to prevent paraffin wax precipitation and deposition, including chemical, mechanical, thermal, and biological treatments with chemical methods being the most effective for improving crude oil flow [11]. Chemical additives offer quick results without major downtime or equipment changes [12, 13]. However, the conventional chemical additives currently employed, such as polyethylene-covinyl acetate (EVA), triethanolamine (TEA), and related polymers, are neither environmentally friendly nor cost-effective [14]. Additionally, conventional paraffin solvents like carbon disulfide are expensive, flammable, and highly toxic when exposed to the environment due to their low flash points [9], while solvent treatments involving hydrophobic chemicals are known for their inflammability, volatility, and high toxicity, posing significant risks to human health and the environment [15, 16].

The preference for cost-effective, eco-friendly wax inhibitors (WIs) promotes the use of plant-based bio-surfactants, which are non-toxic, cheaper to extract, and recoverable during refining. Amino acids are considered eco-friendly due to their biodegradability, non-toxic nature, and environmental safety [17]. These organic compounds are soluble in aqueous media and can be produced at high purity levels at a low cost. Glycine is the simplest amino acid with the chemical formula NH2CH2COOH. It consists of an amine group (-NH2) and a carboxyl group (-COOH) attached to the central carbon atom. It also contains both hydrophilic and hydrophobic regions within its molecular structure [18]. This nature allowed it to adsorb onto the surfaces of wax crystals. The hydrophilic carboxyl group could interact with water molecules present in the crude oil, dispersed the wax particles to smaller sizes. While the hydrophobic methyl group could interact with the non-polar components (long hydrocarbon chains) of the wax crystals. This adsorption prevented the wax crystals from agglomerating and forming large deposits, thereby inhibiting wax deposition.

Another eco-friendly inhibitor is Palm-Based Methyl Ester Sulfonate which can effectively inhibit the formation and deposition of wax crystals in crude oil pipelines and equipment. Its surfactant properties allow its molecules to adsorb onto the surface of wax crystals present in crude oil. By doing so, they modify the surface properties of the wax particles, reducing their tendency to clump together and form large agglomerates. This dispersing action helps to keep the wax particles suspended in the oil phase, preventing them from settling out and depositing on pipeline walls and equipment surfaces, thus maintaining the flowability of crude oil and reducing the risk of blockages. Furthermore, Palm Based Methyl Ester Sulfonates offers an environmentally friendly advantage over certain synthetic alternatives due to its derivation from renewable plant sources.

Currently used conventional inhibitors in the industry significantly increase pipeline operating costs and pose environmental threats due to their toxicity and low biodegradability [19]. This highlights the need for more sustainable and cost-effective alternatives. This study proposes an experimental investigation of eco-friendly chemicals, specifically Amino Acid (Glycine) and Palm-Based Methyl Ester Sulfonate, as inhibitors to mitigate wax precipitation and deposition in crude oil. The research aims to evaluate the effectiveness of these bio-based chemicals in reducing the Wax Appearance Temperature (WAT) and Pour Point Temperature (PPT) of Dulang crude oil and to examine their influence on the crude oil’s rheological characteristics. Limited research has explored the use of such bio-based materials, making this study crucial to addressing the current challenges.

## 2. Materials and methods

### 2.1 Materials

**i. Dulang crude oil.** The samples of crude oil used in the experiment were obtained from the Dulang Oilfield. The physical properties of Dulang crude oil used in the experiments for this project were detailed in Table 1.

**Table 1:** Physical properties of Dulang crude oil [20].

Physical properties	Value	Unit
Density	0.837	g/cm^3^
Specific gravity	0.8378	-
API Gravity	37.39	API
Wax content	28.5	wt%

**ii. Amino Acid–Glycine.** Glycine (NH2CH2COOH), consist of an amine group (-NH2) and a carboxyl group (-COOH) attached to a central carbon atom, gave glycine amphiphilic properties, meaning it had both hydrophilic and hydrophobic regions. The hydrophilic carboxyl group interacted with water molecules in crude oil, while the hydrophobic methyl group interacted with non-polar components of wax crystals, preventing their agglomeration and inhibiting wax deposition. Glycine was provided in powder form. Hence, the inhibitor was prepared by dissolving the glycine powder in distilled water to achieve various concentrations. The concentrations of the wax inhibitor formulated with Glycine were taken as 1%, 5%, and 10%.

**iii. Palm-Based Methyl Ester Sulfonates (MES).** Palm-Based MES is a type of anionic surfactant commonly derived from palm oil derivatives through sulfonation which modified the chemical structure of fatty acids in palm oil. It inhibited wax crystal formation and deposition in crude oil pipelines by adsorbing onto their surfaces, modifying the surface properties of the wax particles, reducing their tendency to clump together and form large agglomerates. This surfactant was supplied by KLK Oleomas Company in Selangor. It contained a mixture of substances with non-hazardous additions consisting of 4016-24-4 Hexadecanoic acid, 2-sulfo-, 1-methyl ester, sodium salt; 4062-78-6 Octadecanoic, 2-sulfo-, 1-methyl ester, sodium salt. It was provided in flake form. Following this, the inhibitor was created by dissolving the MES flake in distilled water to produce different concentrations. The concentrations of the wax inhibitor formulated with MES were taken as 1%, 5%, and 10%.

### 2.2 Apparatuses used

#### i. Cross-Polarized Microscopy (CPM)

The CPM is operated on the principle of polarized light microscopy, where 2 polarizing filters, positioned perpendicular to each other, were used. The first polarizer, positioned below the sample, and the second polarizer is located above the sample which allows light that has been modified by passing through the sample to be observed the crystallization of wax in crude oil.

#### ii. Pour Point Tester (PPT)

The PPT used in this experiment is precisely determined the pour point temperature to within 0.1°C, using the ASTM D5985 standard rotational method. The PPT should be maintained in a stable, temperature-controlled room. The sample cup, the temperature sensor and the sample cup holder should be cleaned from sample remains of former tests to ensure accurate measurements. In this approach, the sample is cooled under constant agitation at 0.1 RPM, allowing the equipment to monitor viscosity changes during cooling. The collected data has been generated as temperature-time profile to accurately identify the pour point based on the average temperature.

#### iii. Rheometer

The rheometer used in this study is used for measuring the rheological properties of materials, such as viscosity (Temperature and Flow Sweep Test, Yield Stress Test). For this experiment, the rheometer applied a constant rotational motion to the sample, measuring the torque required to achieve this rotation at different speeds. The temperature sweep test is conducted at shear rates of 15 1/s while the flow sweep and yield stress test are conducted at 30°C and 20°C with the shear rates varied from 1/s to 200/s. The laboratory environment is maintained at a stable ambient temperature and humidity level to minimize external influences on the measurements.

### 2.3 Experimental procedures

**Fig 1:**
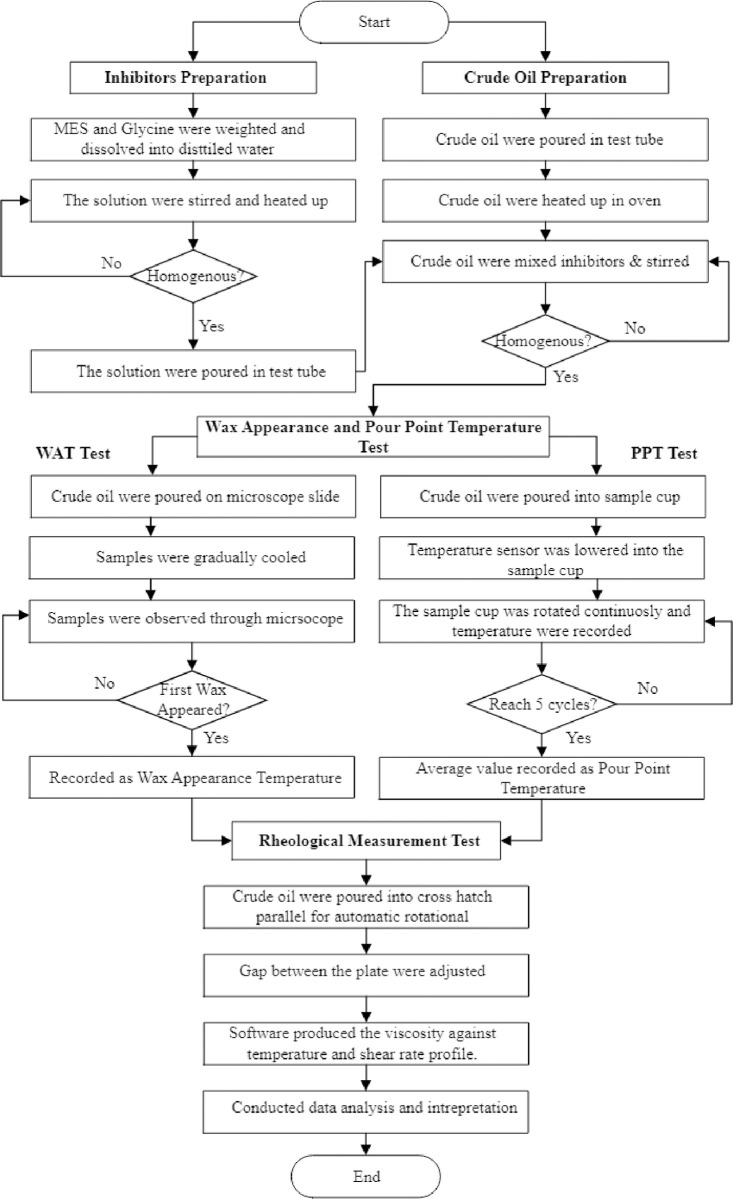
Experimental flowchart.

The experimental flow chart, illustrating and sequence of tests, is shown in Fig 1

#### 2.3.1 Inhibitors preparation

Inhibitors preparation was necessary as both Methyl Ester Sulfonate and Glycine were in flake and powder forms respectively, and the experiment required various concentrations. Therefore, the required mass of inhibitors to prepare these different concentrations was determined depending on the intended concentration. For 1%, 5%, and 10% Glycine and Methyl Ester Sulfonate, the required mass of inhibitors to prepare these different concentrations was determined by dividing the weight of solute (g) by the volume of solution (ml), and found to be 1, 5, and 10 grams of solute (Glycine and MES), respectively. The procedures were conducted as follows: 1 gram of glycine powder was weighed using an electric balance and dissolved in 100 ml of distilled water as a solvent, which was heated to 60°C on a hot plate while stirring continuously using the magnetic stirrer with the constant speed of 500 rpm about 15 minutes to ensure homogeneity. The resulting mixture would likely be clear or slightly cloudy. Then, the solution was divided into three test tubes, each containing 30 ml. This procedure was repeated using 5 grams and 10 grams of glycine, as well as 1 gram, 5 grams, and 10 grams of methyl ester sulfonates.

#### 2.3.2 Crude oil sample preparation

**Fig 2:**
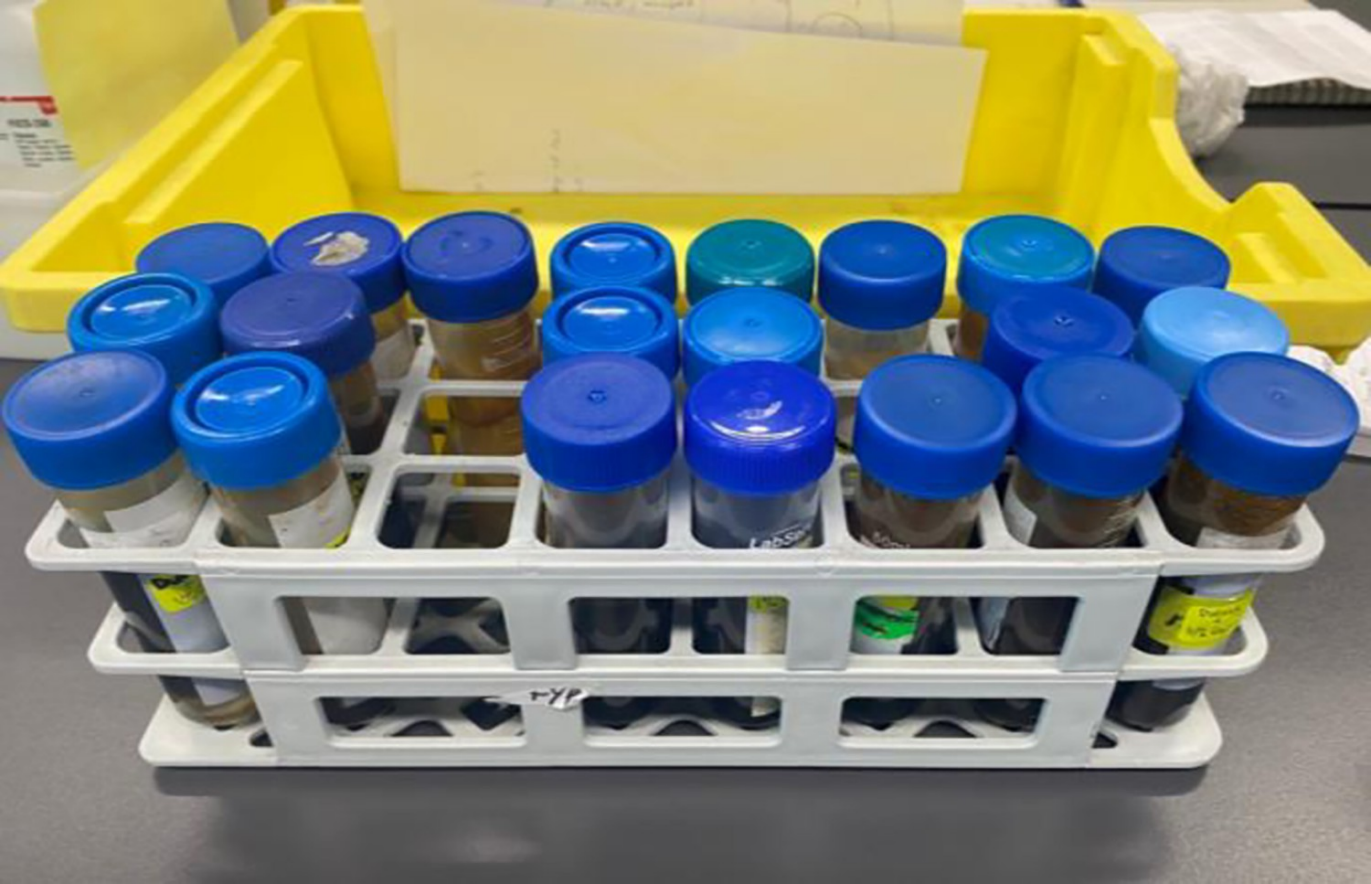
Dulang crude oil in test tube.

Proper sample preparation was essential for accurately investigating the interaction between crude oil and inhibitors at varying concentrations. Preheating the crude oil before experiments ensured complete dissolution of any crystallized wax, addressing potential aging effects from storage or transport. Careful mixing of the crude oil sample with inhibitors according to predefined ratios was crucial. These steps were essential for achieving accurate and representative analysis, forming a reliable basis for evaluating inhibitor effectiveness. The procedure was conducted as follows: Dulang oil was poured into 21 test tubes, each containing 30 ml of the oil, as shown in Fig 2. These test tubes were preheated in a universal oven for approximately half an hour until they reached 60°C to ensure that any pre-crystallized wax in the oil dissolved completely. Subsequently, 10 ml of Glycine, with concentrations of 1%, 5%, and 10%, were added to each 30 ml sample of Dulang crude oil, achieving a 1:3 ratio. The mixtures were then stirred at 60°C for a few minutes until they became homogeneous, promoting the reaction and enhancing the inhibitor’s influence on wax crystals. This process was repeated using palm-based methyl ester sulfonates at the same concentrations of 1%, 5%, and 10%.

#### 2.3.3 Measurements procedure

The procedure of the determination of WAT was as follows:

- A small amount of the crude oil sample was placed on a microscope slide. A cover slip was then applied to spread the sample into a thin layer, ensuring that no air bubbles are present.
- The sample was observed above expected wax appearance temperature using cross-polarized light to establish a baseline appearance of the crude oil sample.
- The sample was gradually cooled. The cooling rate was kept consistent and controlled, around 1°C per minute, to accurately detect the wax appearance temperature.
- The sample was continuously observed through the microscope as it cools. The temperature at which the first wax crystals observed was recorded which was identified as the Wax Appearance Temperature (WAT) for the sample.
- Steps i until iv were repeated with the presence of inhibitors (Glycine and Palm-Based Methyl Ester Sulfonates (1%, 5%, 10%).

The procedure for determining the pour point temperature was conducted as follows: A 30 ml sample of pure crude oil was poured into a container up to the marking line and placed in a container holder. A temperature sensor was then lowered into a telescopic tube and positioned above the sample. The test specified a starting temperature ("Temperature 1") and an ending temperature ("Temperature 2"). The sample was rotated continuously at 0.1 RPM, and this procedure was repeated for five cycles. These steps were then repeated with the addition of inhibitors: Glycine and Palm-Based Methyl Ester Sulfonates at concentrations of 1%, 5%, and 10%.

The procedures for the rheological measurement were conducted as follows: Approximately 4 ml of pure Dulang crude oil was poured into a 40 mm cross hatch parallel plate for an automatic rotational method. The gap between the parallel plates was adjusted to the optimal distance, ensuring consistent sample thickness across all measurements. The recording software was then used to set and produce an analysis profile of viscosity against temperature and shear rate. These steps were repeated with the addition of inhibitors: Glycine and Palm-Based Methyl Ester Sulfonates at concentrations of 1%, 5%, and 10%.

## 3. Results and discussion

### 3.1 Wax appearance temperature

**Fig 3:**
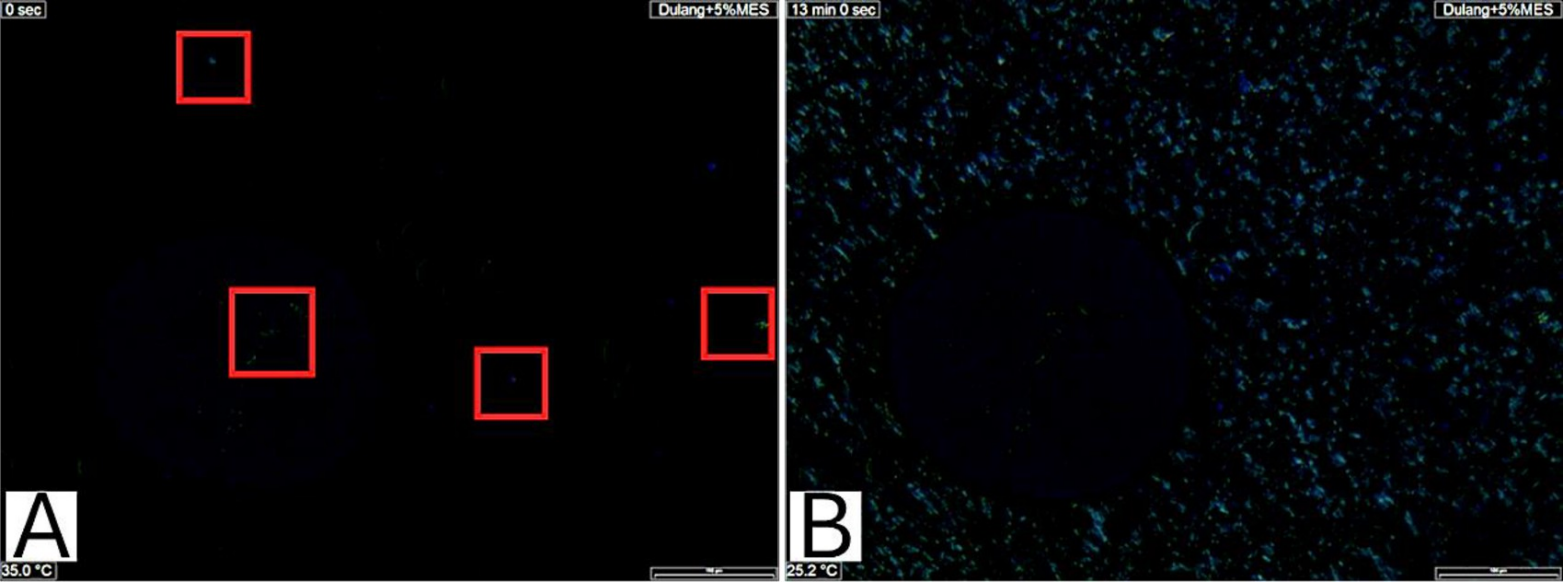
(A) First appearance of wax and (B) Wax precipitation at the end of experiment for Pure Dulang crude oil.

**Fig 4:**
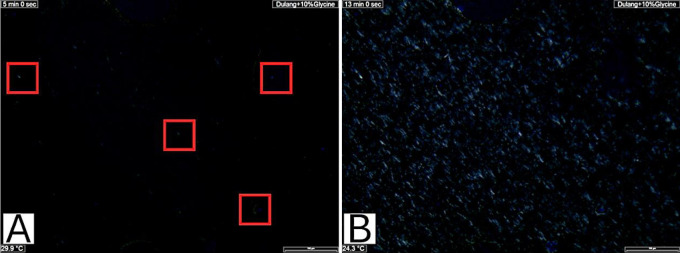
(A) First appearance of wax and (B) Wax precipitation at the end of experiment for Dulang crude oil+10% Glycine.

In this study, the CPM test was employed to observe wax crystal precipitation in Dulang crude oil samples treated with Glycine and MES. Figs 3 and 4 show an example of the images captured at the initial appearance of wax crystals (A), indicating the WAT, and the images at the end of the experiment (B) for pure Dulang crude oil and Dulang crude oil treated with 10% Glycine. Based on the observations of the wax crystalline shape, it was indicated that the wax precipitation primarily consisted of macrocrystalline. This supported the findings of Basem and coworkers [21] which stated that as the temperature decreased, macrocrystalline waxes typically aggregated, forming needle-shaped or flat plate-like crystals. The initial appearance of wax, highlighted within the red boxes in Fig 3A, was observed at 39°C, where faint white wax formations could be seen. In contrast, Fig 3B shows more pronounced needle-shaped wax formations after the temperature was reduced. This Wax Appearance Temperature (WAT) served as an indicator to assess whether the inhibitors could lower the temperature below this point. As shown in Fig 4A, the faint white wax formations in the red boxes were recorded at a WAT of 29.9°C for a 10% concentration of glycine, which is lower compared to the initial WAT.

The WAT from captured images in CPM at different inhibitors and concentrations were summarized in Table 2 below. It was observed that all inhibitors with varying concentrations were able to reduce the WAT of the Dulang crude oil. Glycine, at a concentration of 10%, yielded the highest WAT temperature reduction of all, with a 9.1°C (23.33%) temperature difference from the WAT of untreated Dulang Crude Oil. It was also observed that Glycine was most effective at reducing wax precipitation when used at the highest concentration (10%). However, the effectiveness of the inhibitor did not linearly follow an increase in concentration, as 5% Glycine insignificantly reduced the WAT compared to 1% Glycine. Possible reasons for this non-linear relationship could include scenarios where, at certain concentrations, Glycine competed with other molecules in the solution for binding sites on wax crystals, affecting its overall effectiveness. On the other hand, MES effectively reduced the WAT at the lowest concentration, resulting in a temperature reduction of 8.1°C (20.77%). At higher concentrations, MES might have become less effective in reducing the WAT. This ineffectiveness could have stemmed from MES potentially acting as active sites for wax crystal agglomeration and crystallization. In other words, at higher concentrations, MES might have promoted the formation and growth of wax crystals rather than inhibiting their formation. In this scenario, lower concentrations of MES implied a more conservative usage of MES in the production process, indicating a more cost-effective and sustainable approach to managing wax precipitation in crude oil.

**Table 2:** Wax appearance temperature for different inhibitors and concentration.

Crude oil samples	Wax Appearance Temperature (°C)	Temperature difference (%)
Pure Dulang Crude Oil	39.0	-
Dulang Crude Oil + 1% Glycine	36.0	7.69
Dulang Crude Oil + 5% Glycine	38.4	1.54
Dulang Crude Oil + 10% Glycine	29.9	23.33
Dulang Crude Oil + 1% Methyl Ester Sulfonate	30.9	20.77
Dulang Crude Oil + 5% Methyl Ester Sulfonate	35.0	10.26
Dulang Crude Oil + 10% Methyl Ester Sulfonate	35.0	10.26

In conclusion, the study revealed that 10% Glycine functioned as the most effective inhibitor in reducing the Wax Appearance Temperature (WAT) of crude oil, followed by 1% MES, 5% and 10% MES, 1% Glycine, and lastly 5% Glycine. It was observed that Glycine was less effective at lower concentrations, while MES exhibited the opposite trend. It was demonstrated that the hydrophobic methyl group in Glycine effectively interacted with the non-polar components (long hydrocarbon chains) of the wax crystals. This adsorption interfered with the crystallization process of wax molecules in the crude oil, inhibiting the formation of large wax crystals and thus lowering the temperature at which wax precipitation occurs.

### 3.2 Pour point temperature (PPT)

**Fig 5:**
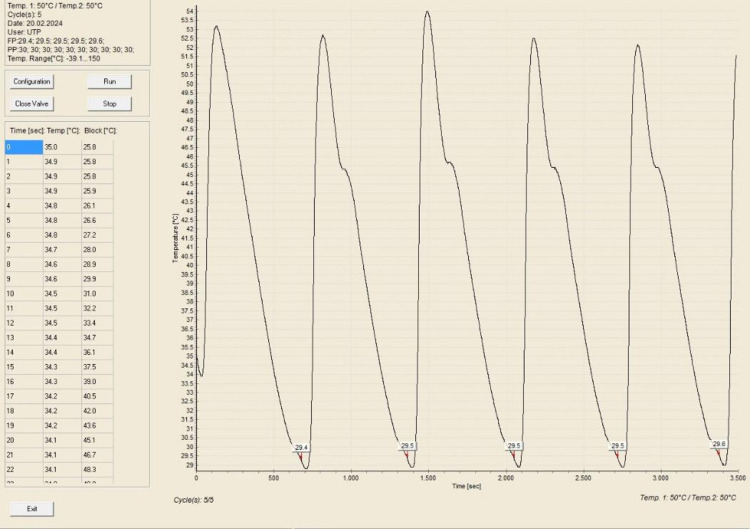
PPT curve for pure Dulang crude oil.

**Fig 6:**
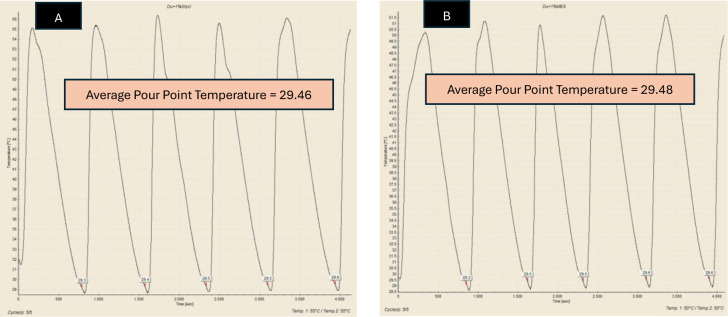
PPT curve for (A) Dulang + 1% Glycine (B) Dulang + 1% MES.

**Fig 7:**
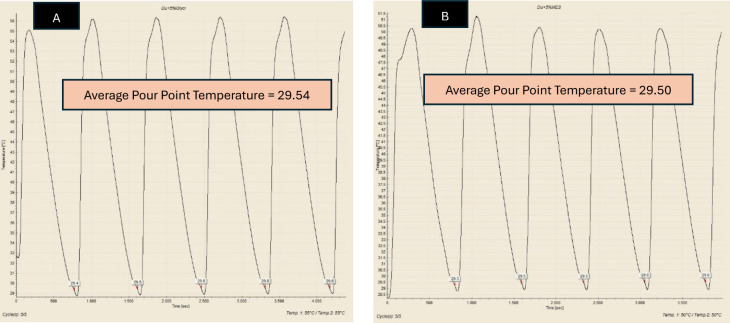
PPT curve for (A) Dulang + 5% Glycine (B) Dulang + 5% MES.

**Fig 8:**
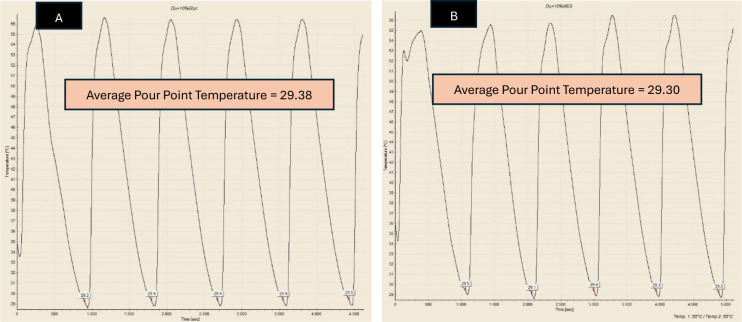
PPT curve for (A) Dulang + 10% Glycine (B) Dulang + 10% MES.

Pour Point Temperature (PPT) is the lowest temperature at which the crude oil would pour or flow under specific conditions. Besides WAT, PPT is also a very important parameter in understanding and managing the behaviour of crude oil in cold environments, as it indicated the point at which oil started to gel or solidify, losing its flowability. In this study, PPT test was employed to observe the reduction of PPT of Dulang crude oil samples treated with Glycine and MES inhibitors. This Pour Point test method involved systematically cooling a sample of crude oil, assessing its flowability as the temperature decreases, followed by a reheating cycle, and the process was repeated for a total of 5 sets. Temperature-time profiles were generated to accurately identify the pour point based on the average temperature. Fig 5 shows the temperature-time profile for pure Dulang crude oil without the presence of inhibitors. This pour point temperature served as a reference to determine whether Glycine and MES could lower the temperature below this point. The pour point temperature recorded for cycle 1, 2, 3, 4 and 5 were 29.4, 29.5, 29.5, 29.5 and 29.6°C, respectively, which yielded the average value of 29.5°C. Part A of Figs 6–8, show the average value of pour point of crude oil when treated with Glycine at 1, 5, and 10% that yielded the value of 29.46, 29.54, and 29.58°C respectively. While part B of Figs 6–8 show the average value of pour point of dulang crude oil when treated with MES at 1, 5, and 10% that yielded the value of 29.48, 29.50, and 29.30° C, respectively.

The temperature-time profile data generated by the Pour Point Tester was summarized in Table 3 below. It can be observed that there were no significant changes in the pour point temperature for any of the treated crude oil samples. It could also be noted that treating crude oil with 5% MES does not lead to any reduction in pour point temperature. On average, Glycine and MES only reduced the temperature by 0.07°C across all concentrations, with the maximum pour point temperature reduction of 0.2°C (0.68%) observed in crude oil treated with 10% MES.

**Table 3:** Average pour point temperature for different inhibitors and concentration.

Crude oil samples	Average Pour Point Temperature (°C)	Temperature Difference (%)
Pure Dulang Crude Oil	29.50	-
Dulang Crude Oil + 1% Glycine	29.46	0.14
Dulang Crude Oil + 5% Glycine	29.54	0.14
Dulang Crude Oil + 10% Glycine	29.38	0.41
Dulang Crude Oil + 1% Methyl Ester Sulfonate	29.48	0.07
Dulang Crude Oil + 5% Methyl Ester Sulfonate	29.50	0
Dulang Crude Oil + 10% Methyl Ester Sulfonate	29.30	0.68

The ASTM D5985 standard method for determining the pour point of petroleum products using a rotational method has been applied. This involved rotating a sample cup filled with crude oil at a slow speed, with a temperature sensor immersed in the fluid. As the temperature dropped, the viscosity increased until the pour point was reached, at which point the increased viscosity moved the sensor, triggering a light barrier to indicate the pour point. Hence, this method had certain limitations, especially when dealing with samples treated with inhibitors. Before performing the pour point test, the crude oil sample was heated to 60°C to ensure that any pre-crystallized wax was completely dissolved. After heating, these water-based inhibitor was added and mixed until homogeneous. While the mixture appeared homogeneous initially, issues occurred when the sample was cooled down in the sample cup for testing. At low temperatures, separation of the mixture occurred, with the inhibitor settling at the bottom and the crude oil layering on top following the density. This separation was crucial because the tiltable bedded temperature sensor, designed to dip into the sample to detect the pour point, could only interact with the separated crude oil layer, not the inhibitor-crude oil enriched portion.

The phenomenon of separation significantly impeded the reliability of pour point measurements, as the detected pour point during the test might not effectively represent the true impact of the inhibitor on the crude oil. Given that the sensor was designed to detect movement attributable to viscosity changes, it primarily interacted with the layer of pure crude oil that had separated from the inhibitor at lower temperatures. As a result, the recorded pour point could either mirror that of untreated crude oil or a fraction of the inhibitor, if any, that remained mixed with the crude oil at the lower temperature, contributing to a small change in the reduction of pour point. Due to this limitation, another round of testing was conducted to observe any differences in PPT. The test was conducted by repeating the same procedure, but the cooling range was reduced (37°C to 0°C), whereas the previous cooling range had been from 56°C to 0°C. The parameter was changed to investigate whether the reduction in cooling range would decrease the phase separation between crude oil and inhibitor, thereby allowing the sensor to detect movements attributable to viscosity changes resulting from the miscible crude oil and inhibitor.

**Fig 9:**
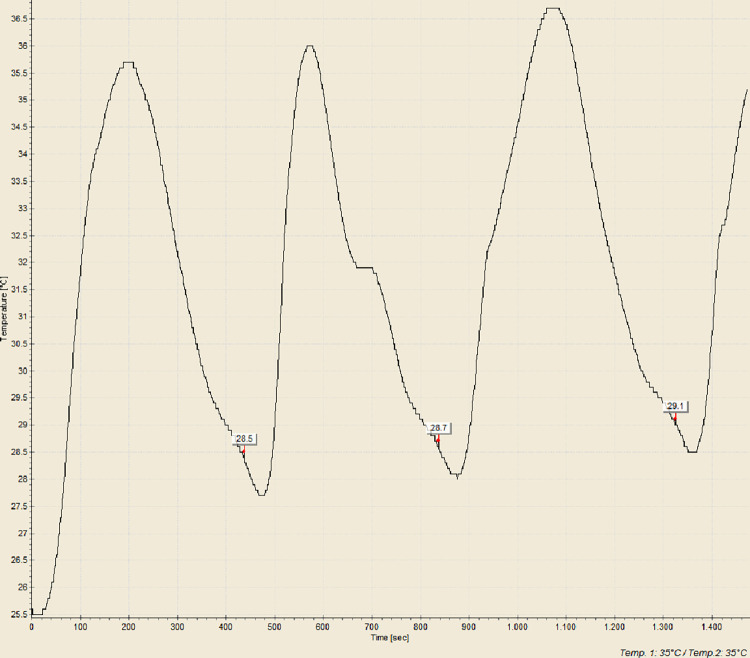
PPT curve for Dulang crude oil+10% Glycine.

Fig 9 show the results for the repeated procedure on the PPT curve for Dulang crude oil treated with 10% Glycine. It could be seen that the pour point temperature recorded for cycles 1, 2, and 3 was 28.5, 28.7, and 29.1°C, respectively, yielding an average value of 28.7°C. However, the procedure also produced the same insignificant PPT results. Since the result showed insignificant reduction in PPT, both MES and Glycine can be justified and recommended to be used as flow improvers to enhance flowability during pipeline operation and not recommended to be used as pour point depressants (PPDs).

### 3.3 Rheological measurement (viscosity)

Rheological measurements using rheometer, including Temperature Sweep and Flow Sweep tests, were conducted to study the behavior of Dulang crude oil with Glycine and MES as inhibitors. These tests aimed to understand how the addition of inhibitors affected the flow properties of crude oil under various conditions.

#### 3.3.1 Temperature sweep

**Fig 10:**
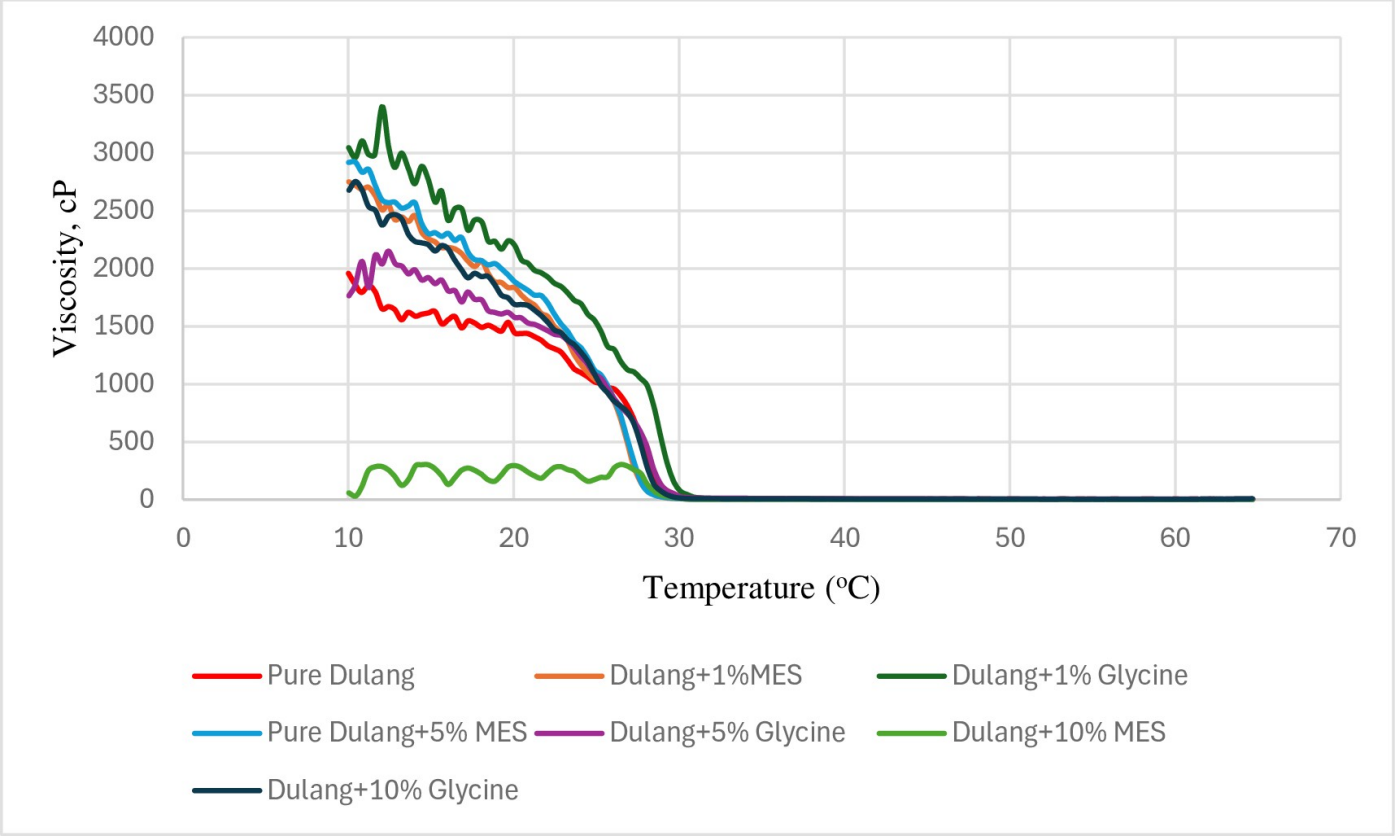
Temperature sweep (cooling) for Dulang crude oil with inhibitors.

In this study, a temperature sweep test involved cooling and heating of crude oil from high temperatures to lower temperatures and vice versa, where wax began to precipitate. Generally, as the temperature decreased below the WAT, crude oil tended to show transition from Newtonian to non-Newtonian behaviour, resulting in significant increases in viscosity. This transition point is referred to as the abnormal point, marking the division between non-Newtonian and Newtonian shear-thinning behaviours. Inhibitors proved their effectiveness by their ability to reduce viscosity at low temperatures within the range of non-Newtonian behaviour. Therefore, further interpretation of the viscosity-temperature profile was conducted to confirm the success of inhibitors in reducing the viscosity of crude oil regarding the effect of temperatures from 10 to 64°C and shear rates (15 1/s). It was observed that the viscosity increased significantly when the temperature decreased to 10°C. When dulang crude oil was treated with MES (1, 5, and 10%) as shown in Fig 10, it was noted that the crude oil changed its shear-thinning behaviour from Newtonian to non-Newtonian at a lower temperature compared to the untreated crude oil. However, only the 10% MES treatment resulted in reducing the significant rise in viscosity when subjected to further temperature decrease from 30°C to 10°C. Next, when crude oil was treated with Glycine in Fig 10, only 5 and 10% Glycine were able to change the crude oil behaviour to non-Newtonian at a lower temperature than the untreated crude oil. It was concluded that the most effective inhibitor in reducing the significant rose in crude oil viscosity within the range of non-Newtonian behaviour was achieved by 10% MES treatment.

**Fig 11:**
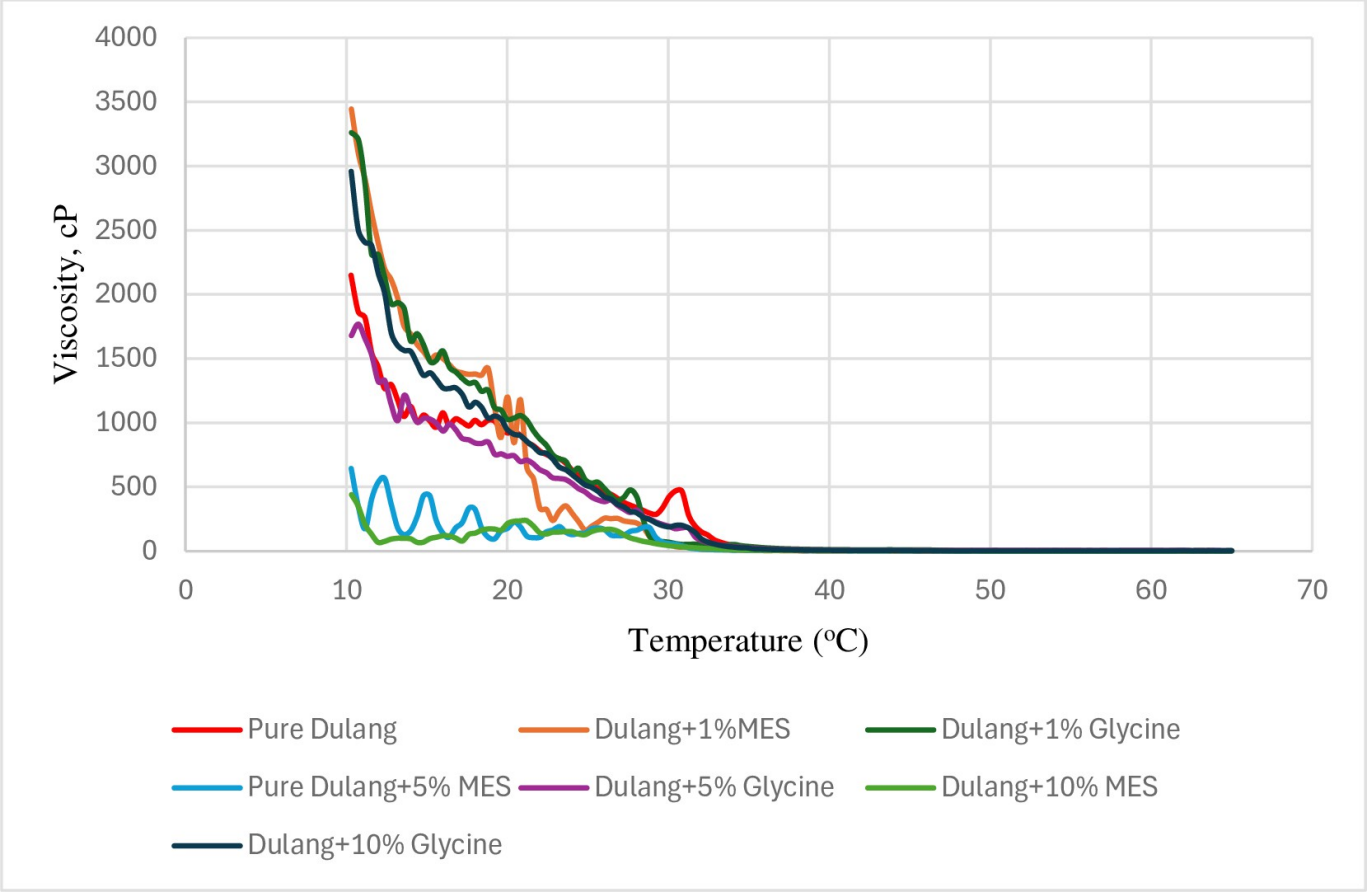
Temperature sweep for (heating) Dulang crude Oil+Inhibitors.

Next, the flow behaviour of crude oil with the presence of inhibitors under the influence of varying temperature was further evaluated by conducting the temperature sweep in heating order. It showed that the viscosity decreased significantly when the temperature increased to 64°C attributed to the dissolution of wax in the crude oil. When dulang crude oil was treated with MES (1, 5, and 10%), it showed that crude oil changed its behaviour from non-Newtonian to Newtonian at lower temperature compared to the untreated crude oil as shown in Fig 11. However, only 5 and 10% MES able to decrease the viscosity when subject to further increase in temperature. Next, when crude oil was treated with Glycine, the inhibitor was able to change the crude oil behaviour to Newtonian at lower temperature than untreated crude oil. However, only 5% Glycine was able to decrease the viscosity when subject to further increase in temperature. From Fig 11, 1% MES, 5 and 10% Glycine reduced the viscosity but not lower than the reduction in pure crude oil. It could also be concluded that 10% MES has the highest effectiveness in reducing the viscosity of crude oil within the range of non-Newtonian behaviour, following the same trend in cooling order.

The use of MES and Glycine resulted in a change in behaviour from Newtonian to non-Newtonian at lower temperatures compared to untreated crude oil, showing the improvement of its flow characteristics and viscosity profile. The transition from Newtonian to non-Newtonian behaviour suggests that the treated crude oil was better able to maintain its flowability and resistance to viscosity increase, even at lower temperatures where wax precipitation would typically cause significant viscosity increase. In summary, as crude oil is cooled and further dropped below the WAT, an increased in viscosity occurred due to the formation of plate-like wax crystals within the crude oil. The efficacy of 10% MES in reducing viscosity below that of pure crude oil was demonstrated, highlighting its success. This proved that 10% MES successfully disrupted the wax crystal formation in crude oil, interfering with wax molecule aggregation and thereby preventing the formation of large, interconnected networks that increased crude oil viscosity.

#### 3.3.2 Flow sweep

**Fig 12:**
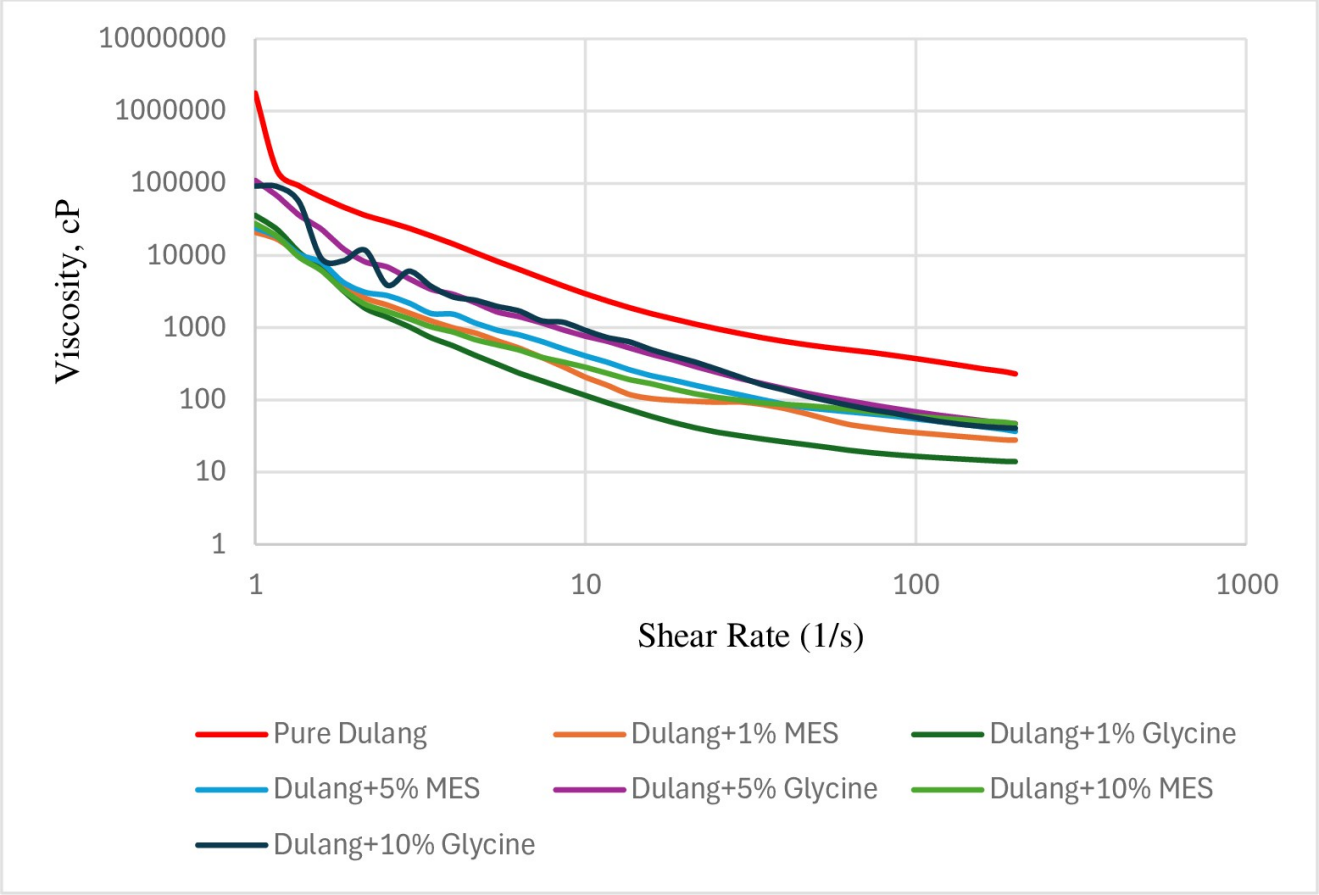
Flow sweep test for Dulang crude Oil + Inhibitors at 20°C.

**Fig 13:**
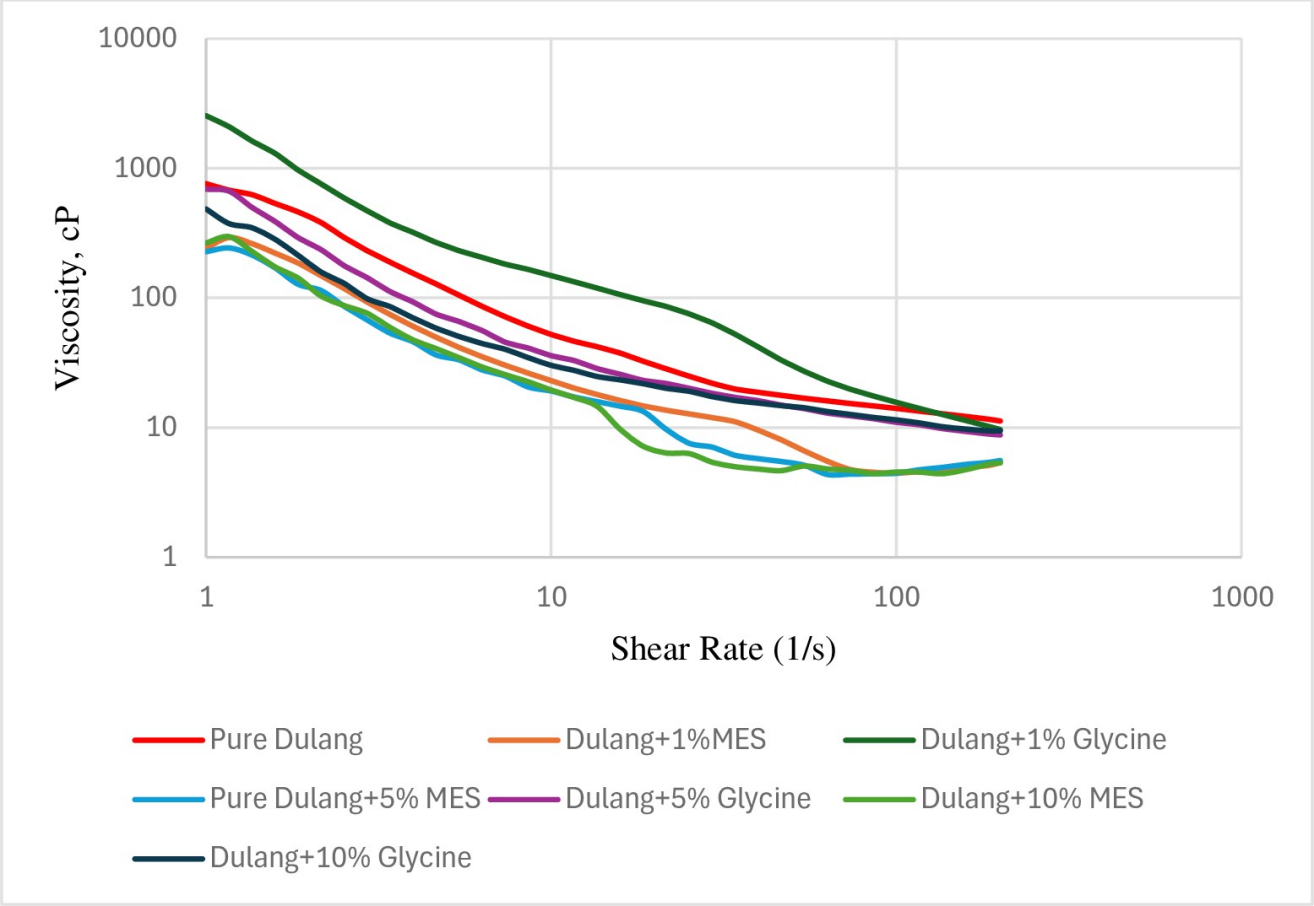
Flow sweep test for Dulang crude Oil + Inhibitors at 30°C.

In this study, a flow sweep tests were conducted for additional investigation to prove the effectiveness of MES and Glycine in reducing crude oil viscosity across a range of shear rates. Viscosity-shear rate tests were conducted at 30°C and 20°C, which at PPT and below PPT respectively. The shear rates varied from 1/s to 200/s. The relationship between viscosity and shear rate for both untreated and treated crude oil was studied in Figs 12 and 13, where viscosity was observed to decrease continuously as shear rate increased. At 20°C, it was found that both MES and Glycine were able to reduce the viscosity of the crude oil across various shear rates. Specifically, 1% Glycine was the most effective in reducing viscosity, followed by 1% MES, 10% MES, 5% MES, 5% Glycine, and 10% Glycine, respectively. At 30°C, both MES and Glycine were observed to reduce the viscosity of the crude oil across varying shear rates, except for 1% Glycine, as shown in Fig 13. In this case, 10% MES was found to be the most effective in viscosity reduction, followed by 5% MES, 1% MES, 10% Glycine, and then 5% Glycine. Additionally, it could also be observed that as the shear rate further increased, the viscosity reduction continued until it reached a constant value, suggesting that a steady equilibrium state had been achieved. Therefore, the decrease in viscosity was smaller at the maximum shear rate of 200/s.

The effectiveness of Glycine and MES depended on their ability to interact with the crude oil components and alter its rheological properties. At 20°C, which is below the pour point of the crude oil, 1% glycine was the most effective inhibitor that reduced viscosity. While 10% MES still effectively reduced viscosity like how it worked in temperature sweep test, but not as effective as 1% Glycine. Glycine more effectively disrupted the crystalline structure of wax molecules in the crude oil compared to MES, preventing them from forming large aggregates that contributed to higher viscosity. Since the temperature was below the pour point, the wax molecules were already beginning to solidify, and glycine helped inhibit further crystallization, allowing the oil to flow more easily. However, the effectiveness of 1% glycine might have degraded, as it did not work in reducing viscosity at higher temperatures and shear rates. At 30°C, high concentration of MES effectively interacted with the crude oil components, improving its flow behaviour by reducing viscosity and enhancing shear thinning properties. Additionally, at higher temperatures, the viscosity of the crude oil tended to decrease, and MES could further facilitate this reduction by modifying the oil’s rheological properties, making it more effective.

Next, further rheological measurements were studied regarding the yield stress of the untreated and treated of dulang crude oil with MES and Glycine. Table 4 summarized the influence of treated Dulang crude oil on the yield stress values below the pour point (20°C) and at the pour point temperature (30°C). In the first case, the untreated crude oil exhibited a stress value of 0.63 at 30°C and 48.86 at 20°C. The yield stress was higher at 20°C because below pour-point, as the temperature decreases, more waxes solidified, thus contributed to an increase in the yield stress. These solidified waxes acted as a network that must be overcome for the oil to flow, thus requiring a higher stress. So, as the temperature decreases below the pour point, the viscosity of the crude oil increased. This increase in viscosity was due to the decrease in thermal energy, which reduced the mobility of the oil molecules. Higher viscosity contributed to a higher yield stress because more force was required to overcome the resistance to flow.

**Table 4:** Yield value at 30°C and 20°C.

Sample	Yield Value (Pa)	
	At 30°C	At 20°C
Pure Dulang	0.63	48.86
Dulang+1% Glycine	1.57	1.47
Dulang+5% Glycine	0.44	7.29
Dulang+10% Glycine	0.31	6.66
Dulang+1% MES	0.28	3.05
Dulang+5% MES	0.21	4.65
Dulang+10% MES	0.2	4.15

When crude oil was treated with inhibitors at various concentrations, observations revealed that at the pour point (30°C), both glycine and MES effectively lowered the yield stress, except for 1% glycine. Notably, MES at 10% exhibited the most significant reduction in yield stress, decreasing it by approximately 0.43 Pa compared to untreated crude oil. Below pour point (20°C), both glycine and MES were able to reduce the yield stress. Glycine at 1% reduced the highest value of yield stress by about 47.39 Pa compared to untreated crude oil. In summary, the results demonstrated a consistent trend with the viscosity-shear rate profile. 1% Glycine proved to be the most effective inhibitor at 20°C, while 10% MES at 30°C respectively. Last but not least, the second objective of this study, aimed at investigating the influence of bio-based chemical inhibitors on the rheological characteristics of crude oil, was successfully achieved.

## 4. Conclusions

Two bio-based chemical inhibitors, Glycine and Palm-Based Methyl Ester Sulfonate (MES) were used to formulate an environmentally sustainable solution. Glycine, sourced from amino acids, and MES, derived from plant oils, are both clean and eco-friendly. Experimental results revealed that both Glycine and MES effectively reduced the wax appearance temperature (WAT), viscosity, and yield point of waxy crude oils. Specifically, 10% Glycine was the most effective, reducing WAT by 23.3%. However, across all concentrations studied (1%, 5%, and 10%), MES demonstrated greater overall effectiveness, with an average WAT reduction of 13.76% compared to Glycine’s 10.85%. Rheological tests showed a similar trend: while 1% Glycine efficiently reduced viscosity and yield point at low temperatures, MES was more effective on average in reducing both viscosity and yield point. This suggests that MES is generally the better inhibitor for mitigating paraffin deposition in an environmentally friendly manner. Since pour point temperature (PPT) results were insignificant due to limitations, MES was recommended as a flow improver rather than a pour point depressant (PPD). Ultimately, the development of these newly formulated chemical inhibitors promises a greener and more economically efficient approach to maximizing production from mature oil fields.

## Supporting information

S1 Data (ZIP)
