# Supplementary figures and images for: Improving paraffin precipitate inhibition using glycine and Palm-based Methyl Ester Sulfonate (MES) eco-friendly inhibitors
Source: PLoS One. 2025 Jan 28;20(1):e0313394. doi: 10.1371/journal.pone.0313394 (PMC11774394; doi:10.1371/journal.pone.0313394)

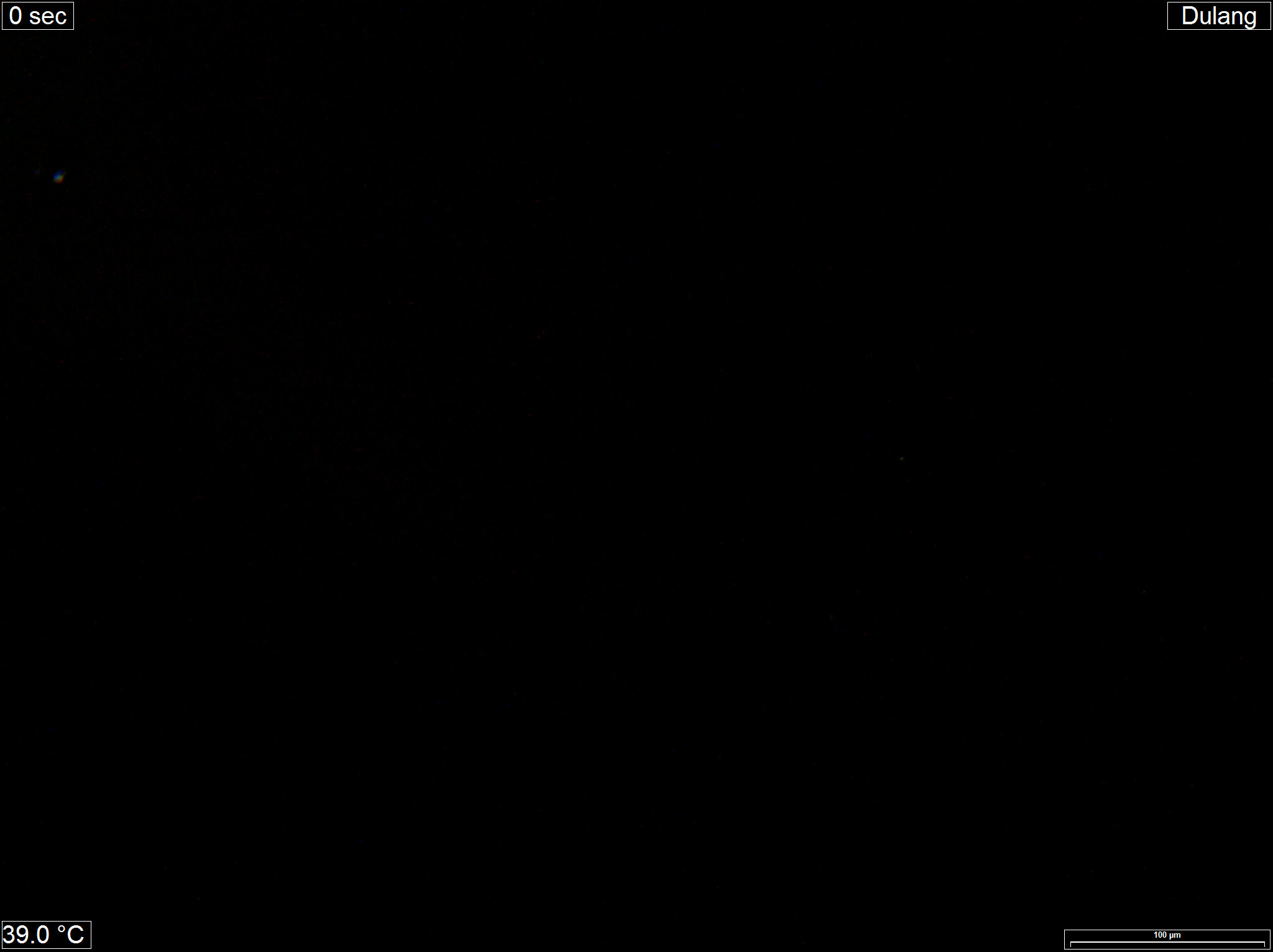

Supplement: S1 Data — (ZIP) [file pone.0313394.s001.zip › Data/CPM/Dulang_001.tif]

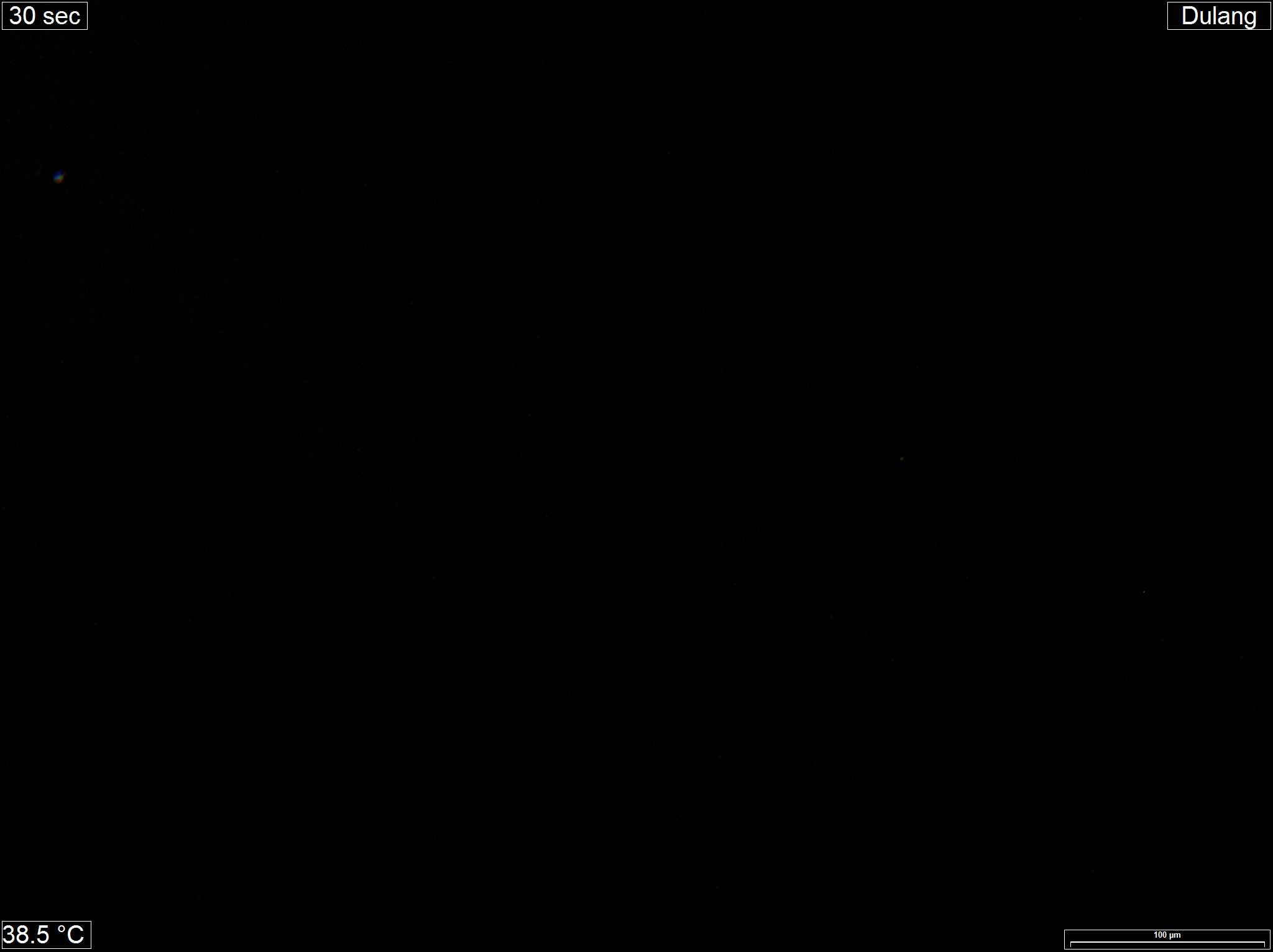

Supplement: S1 Data — (ZIP) [file pone.0313394.s001.zip › Data/CPM/Dulang_002.tif]

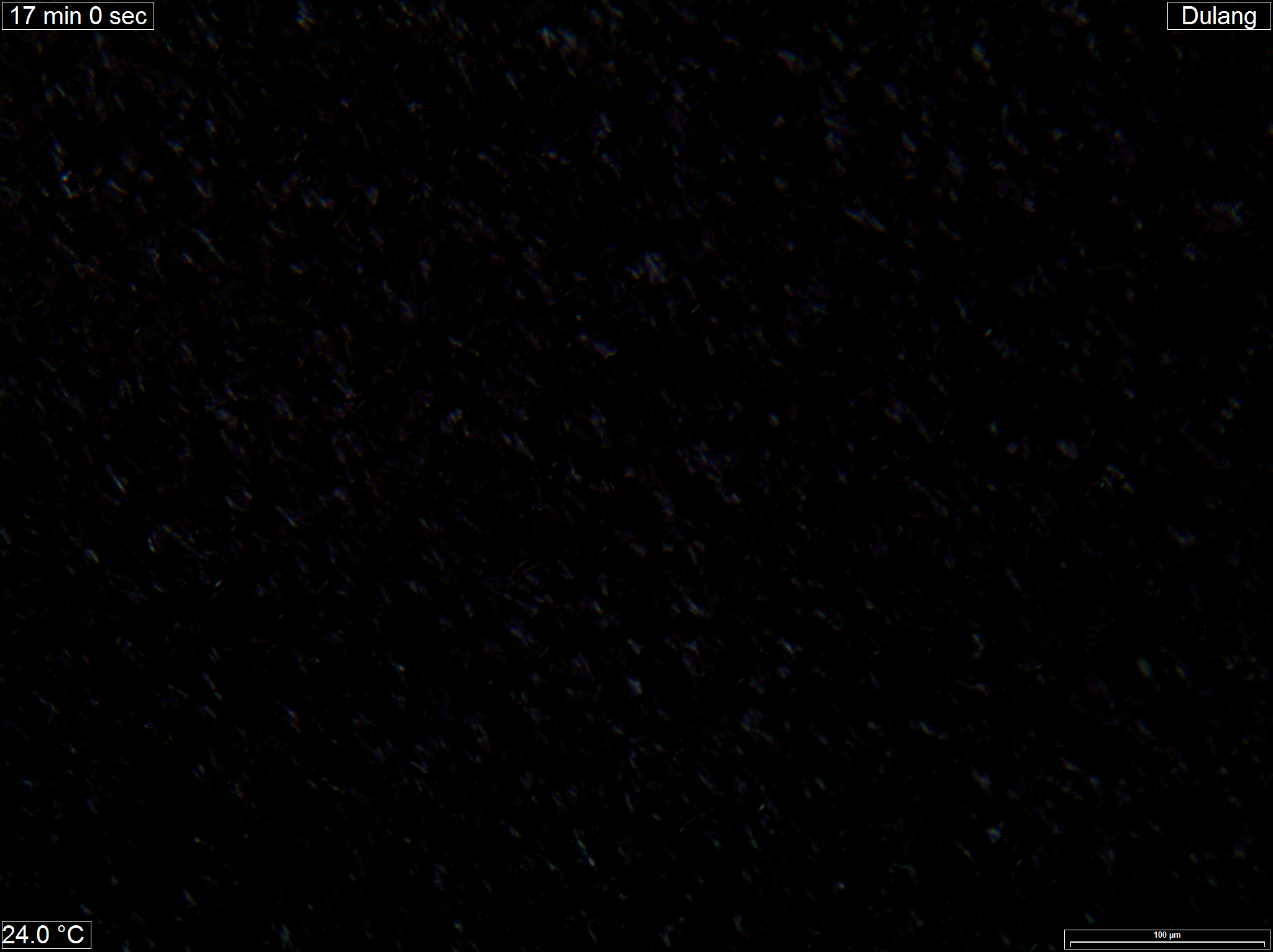

Supplement: S1 Data — (ZIP) [file pone.0313394.s001.zip › Data/CPM/Dulang_035.tif]

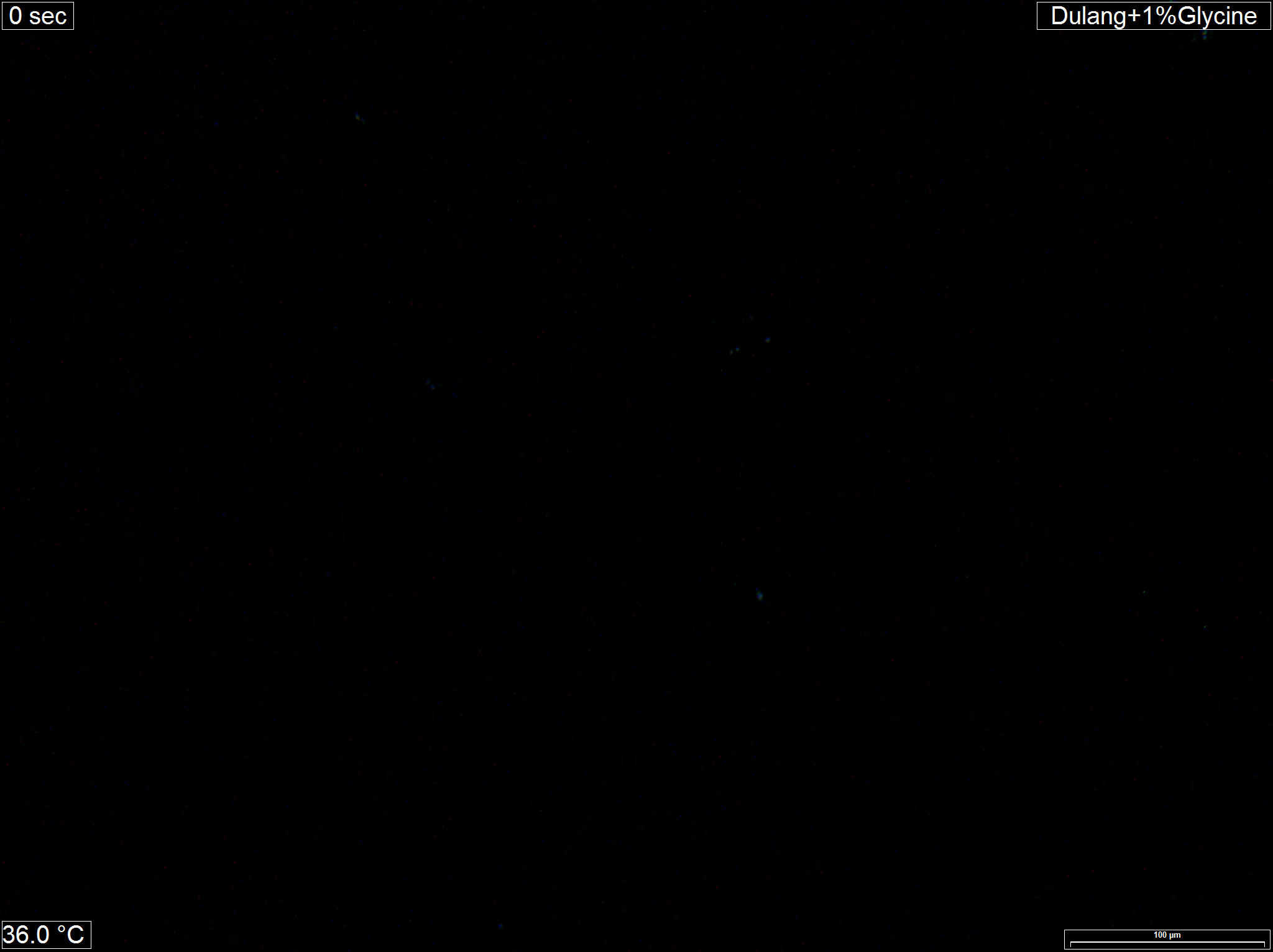

Supplement: S1 Data — (ZIP) [file pone.0313394.s001.zip › Data/CPM/Dulang+1%Glycine_001.tif]

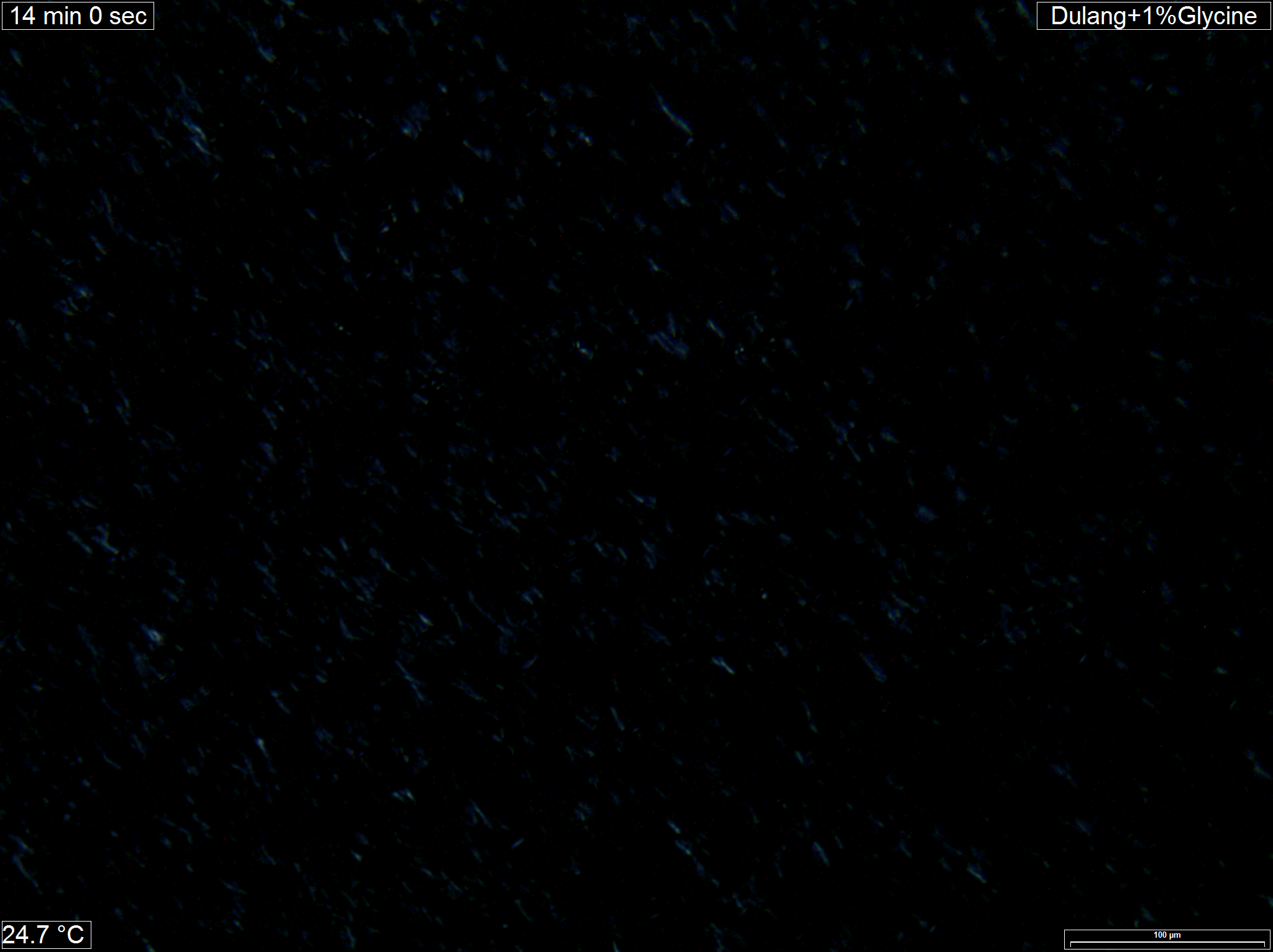

Supplement: S1 Data — (ZIP) [file pone.0313394.s001.zip › Data/CPM/Dulang+1%Glycine_029.tif]

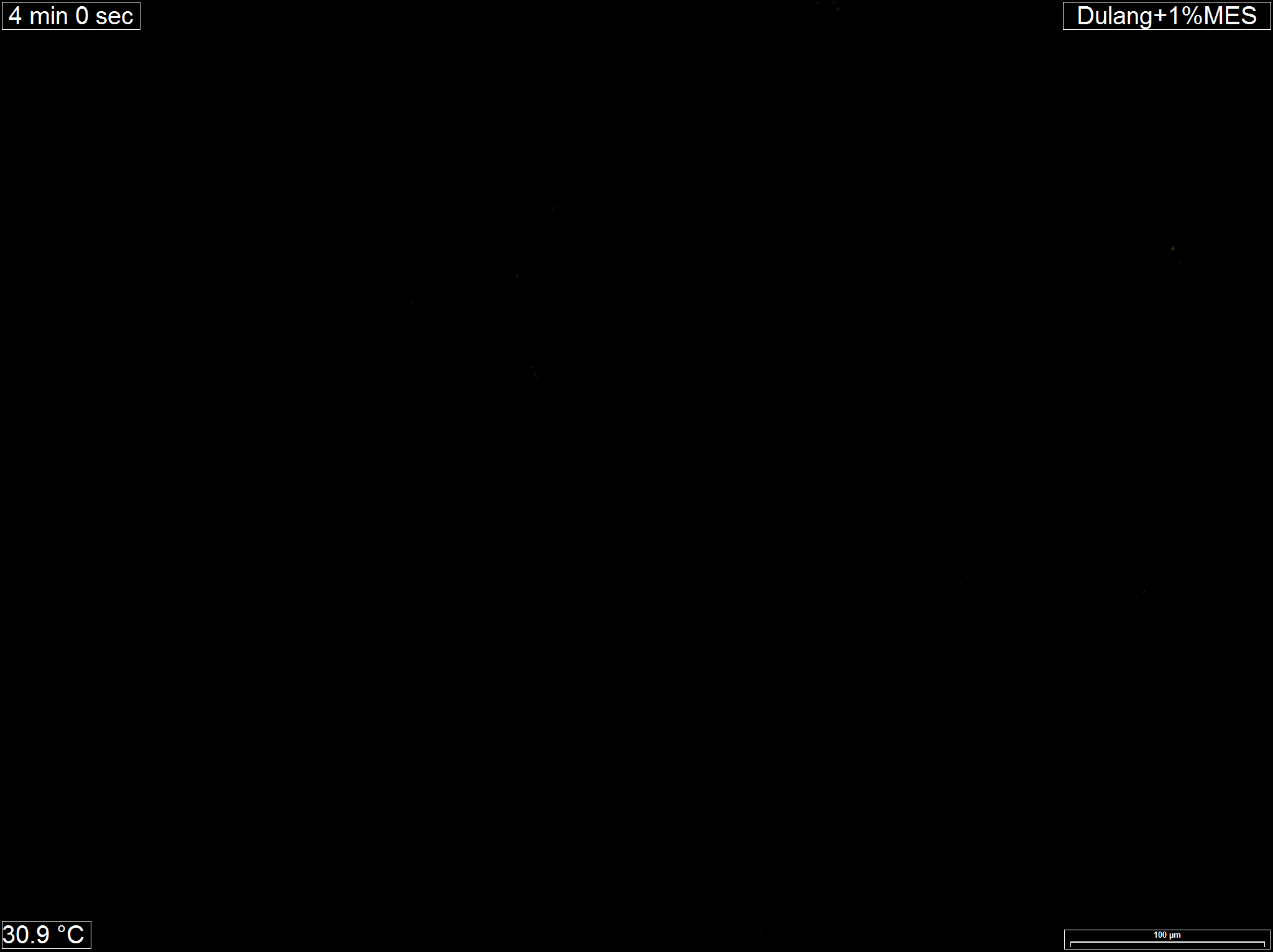

Supplement: S1 Data — (ZIP) [file pone.0313394.s001.zip › Data/CPM/Dulang+1%MES_009.tif]

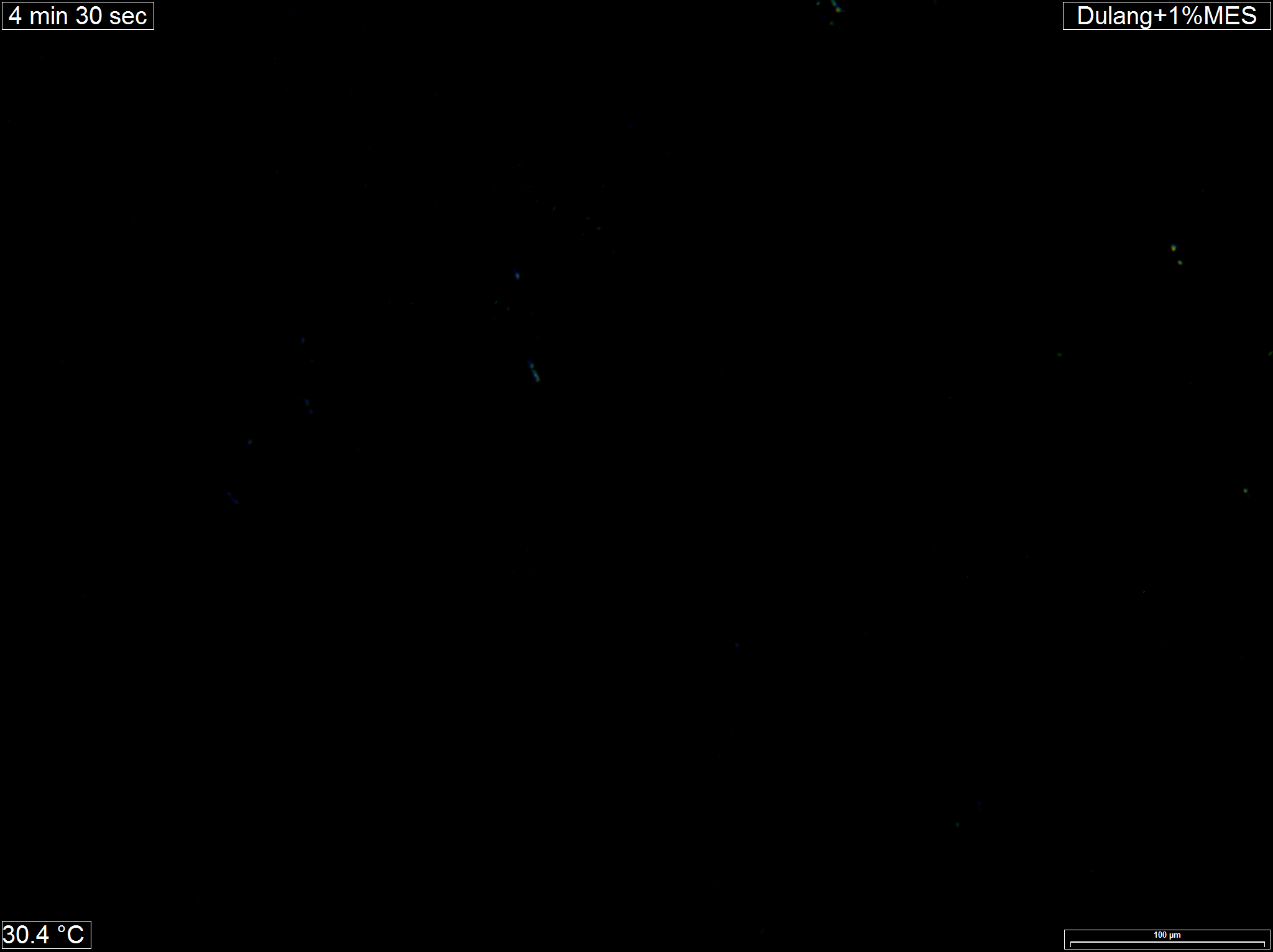

Supplement: S1 Data — (ZIP) [file pone.0313394.s001.zip › Data/CPM/Dulang+1%MES_010.tif]

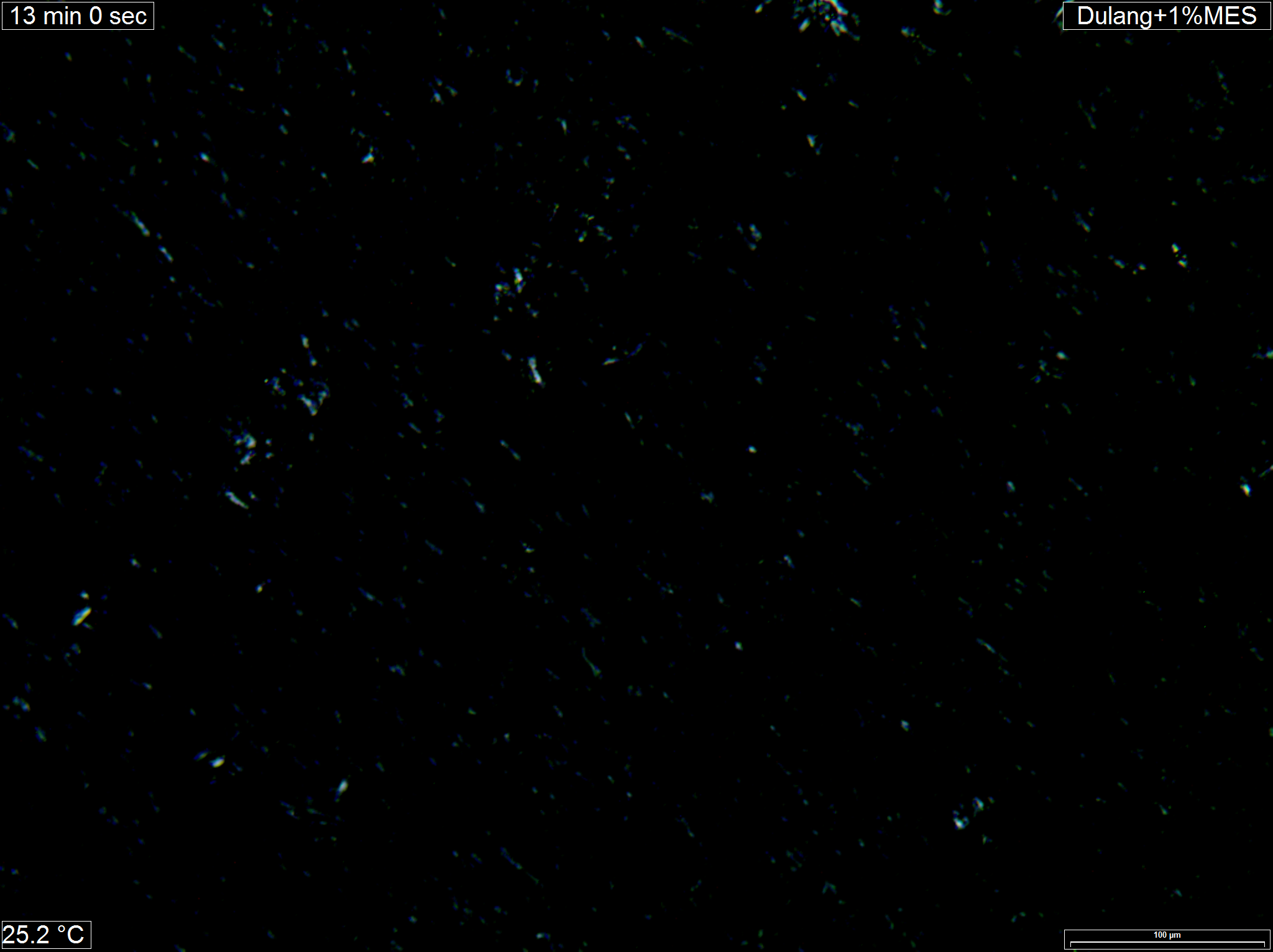

Supplement: S1 Data — (ZIP) [file pone.0313394.s001.zip › Data/CPM/Dulang+1%MES_027.tif]

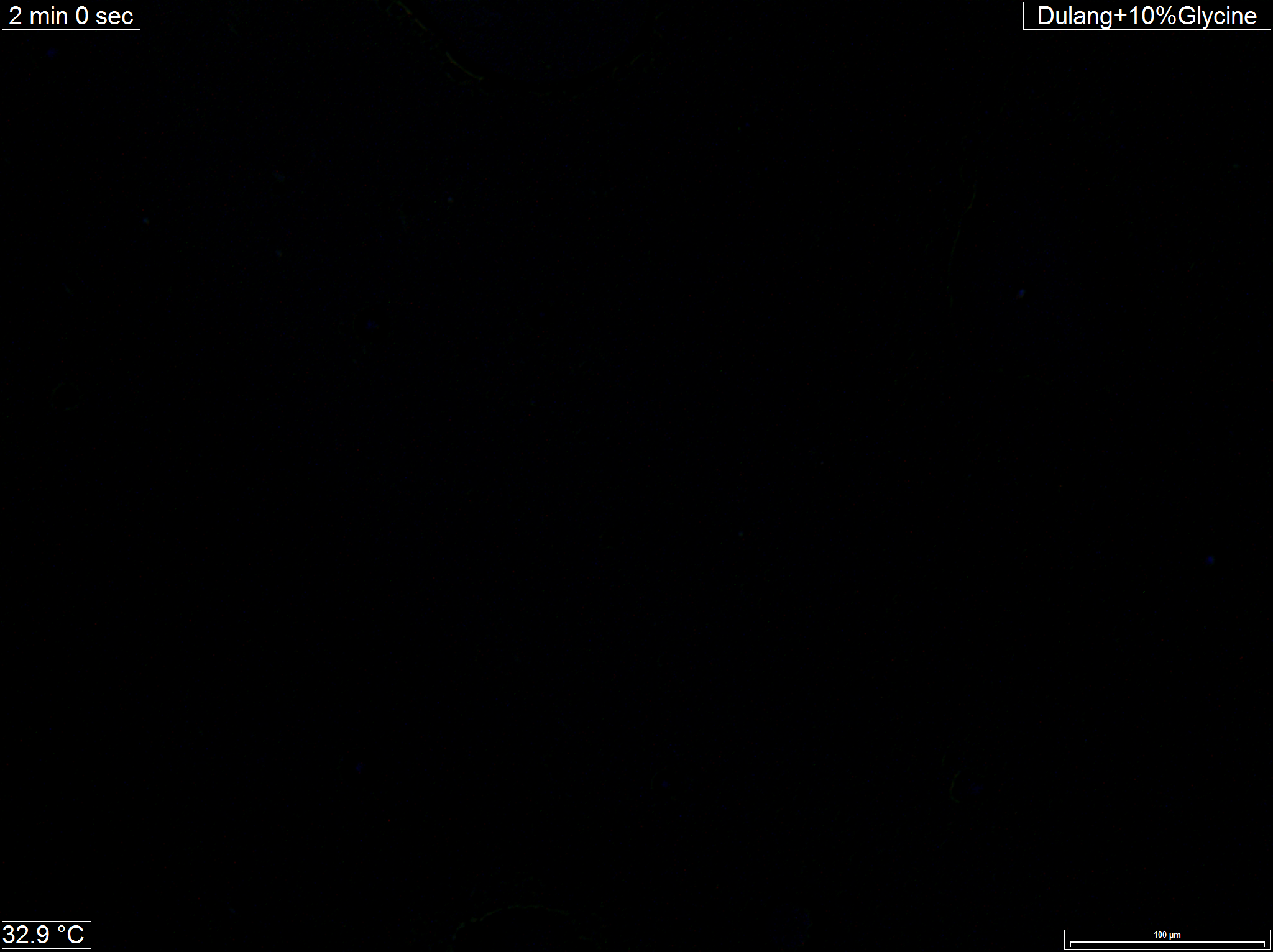

Supplement: S1 Data — (ZIP) [file pone.0313394.s001.zip › Data/CPM/Dulang+10%Glycine_005.tif]

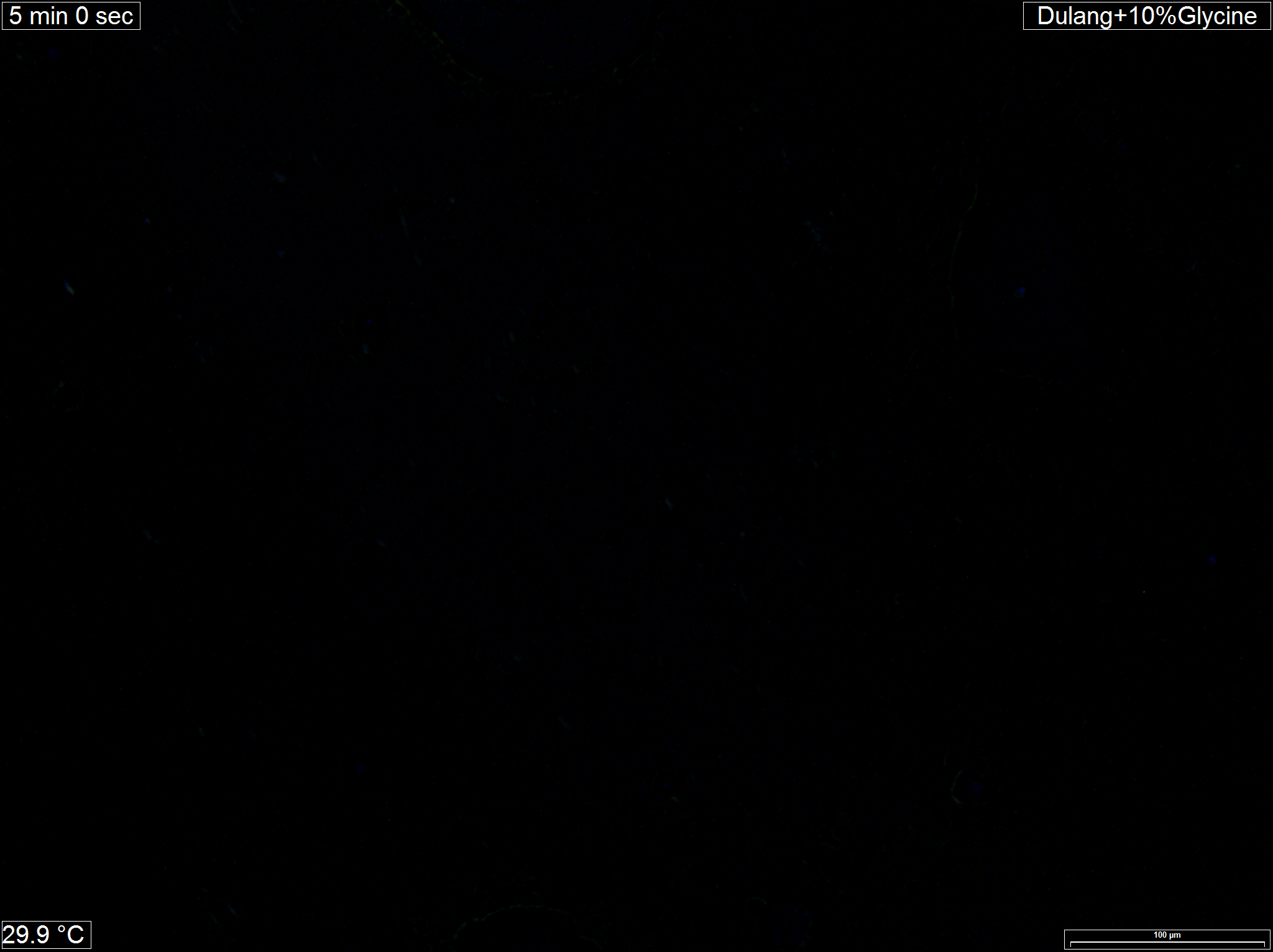

Supplement: S1 Data — (ZIP) [file pone.0313394.s001.zip › Data/CPM/Dulang+10%Glycine_011.tif]

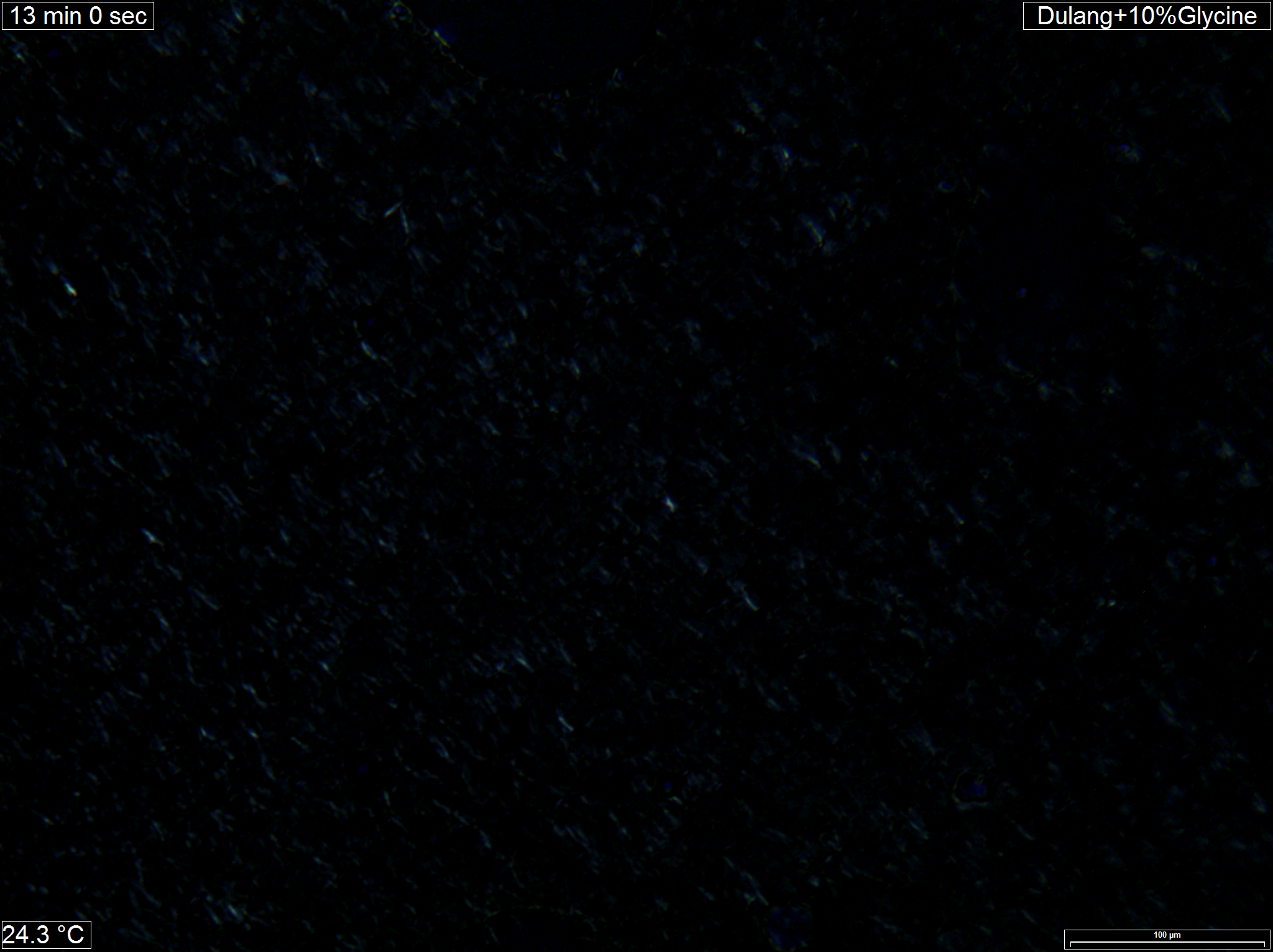

Supplement: S1 Data — (ZIP) [file pone.0313394.s001.zip › Data/CPM/Dulang+10%Glycine_027.tif]

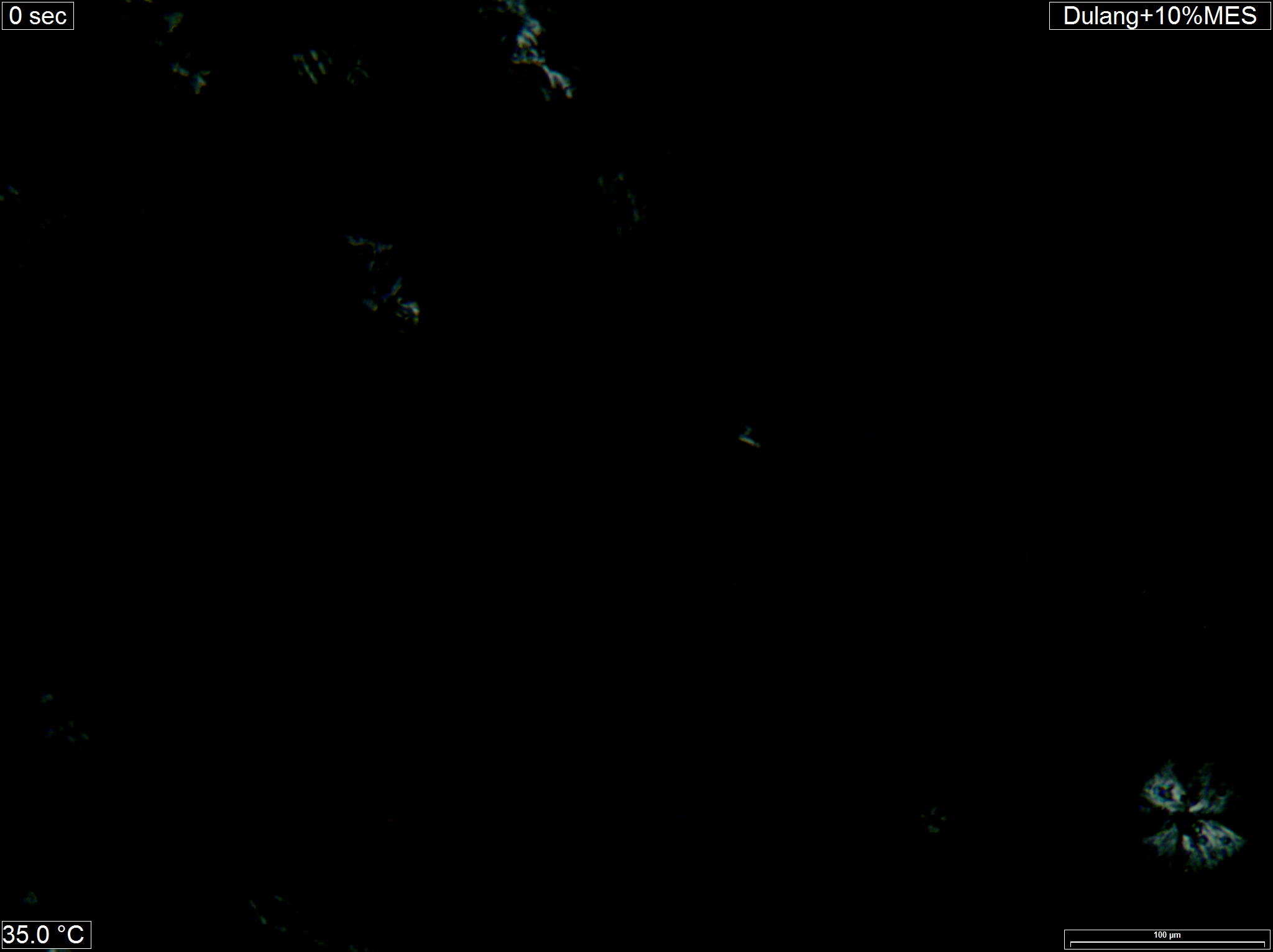

Supplement: S1 Data — (ZIP) [file pone.0313394.s001.zip › Data/CPM/Dulang+10%MES_001.tif]

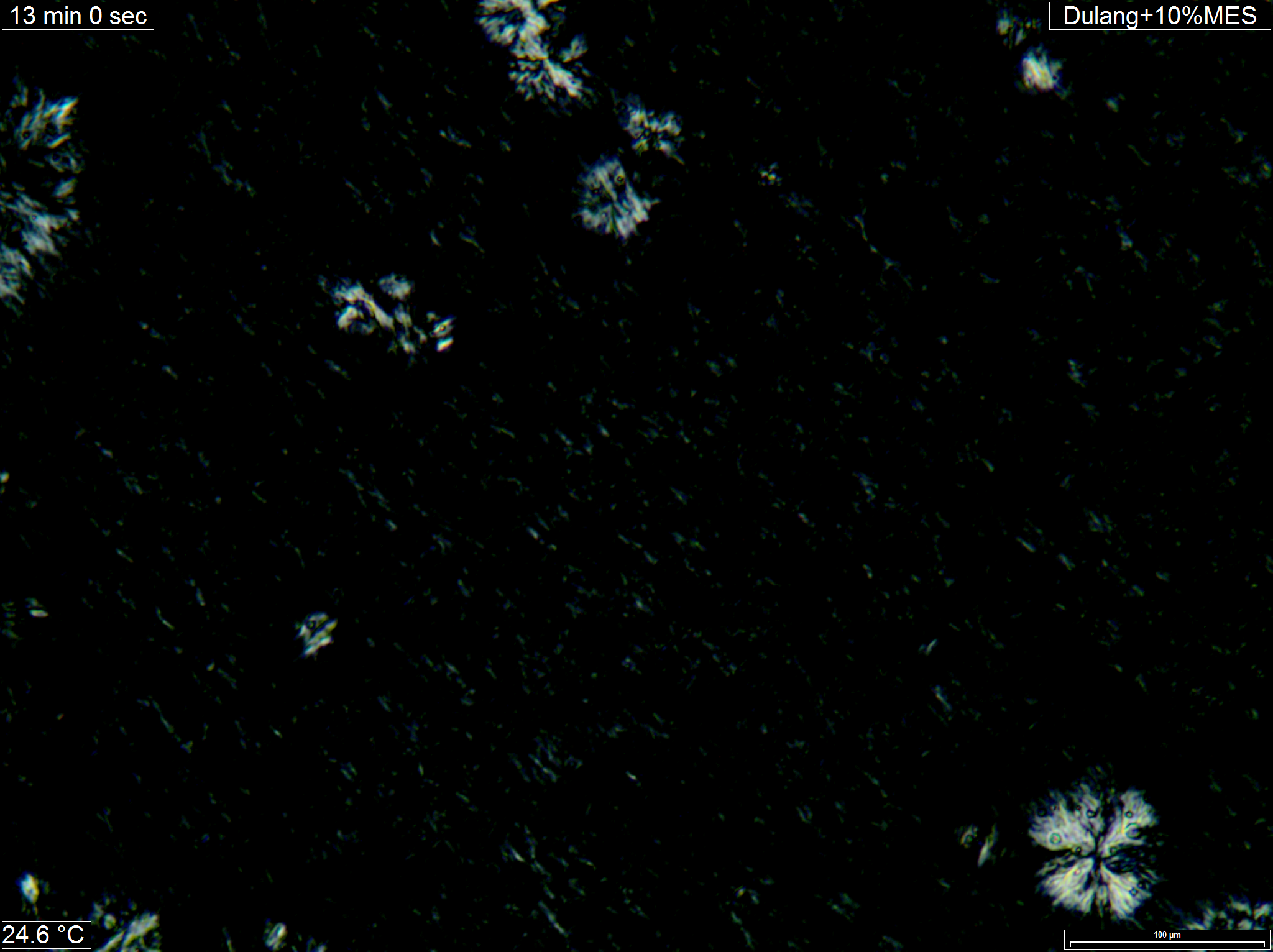

Supplement: S1 Data — (ZIP) [file pone.0313394.s001.zip › Data/CPM/Dulang+10%MES_027.tif]

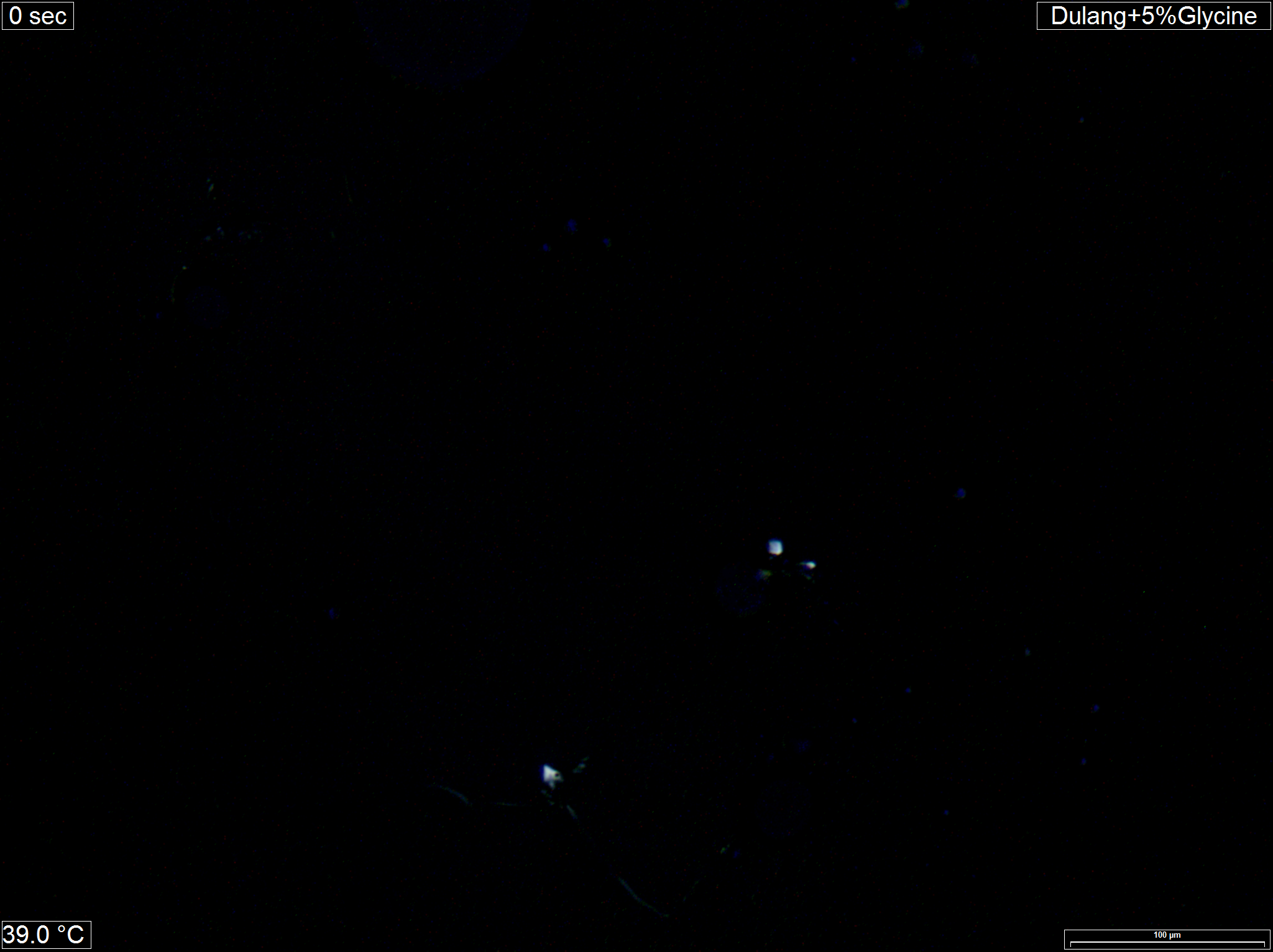

Supplement: S1 Data — (ZIP) [file pone.0313394.s001.zip › Data/CPM/Dulang+5%Glycine_001.tif]

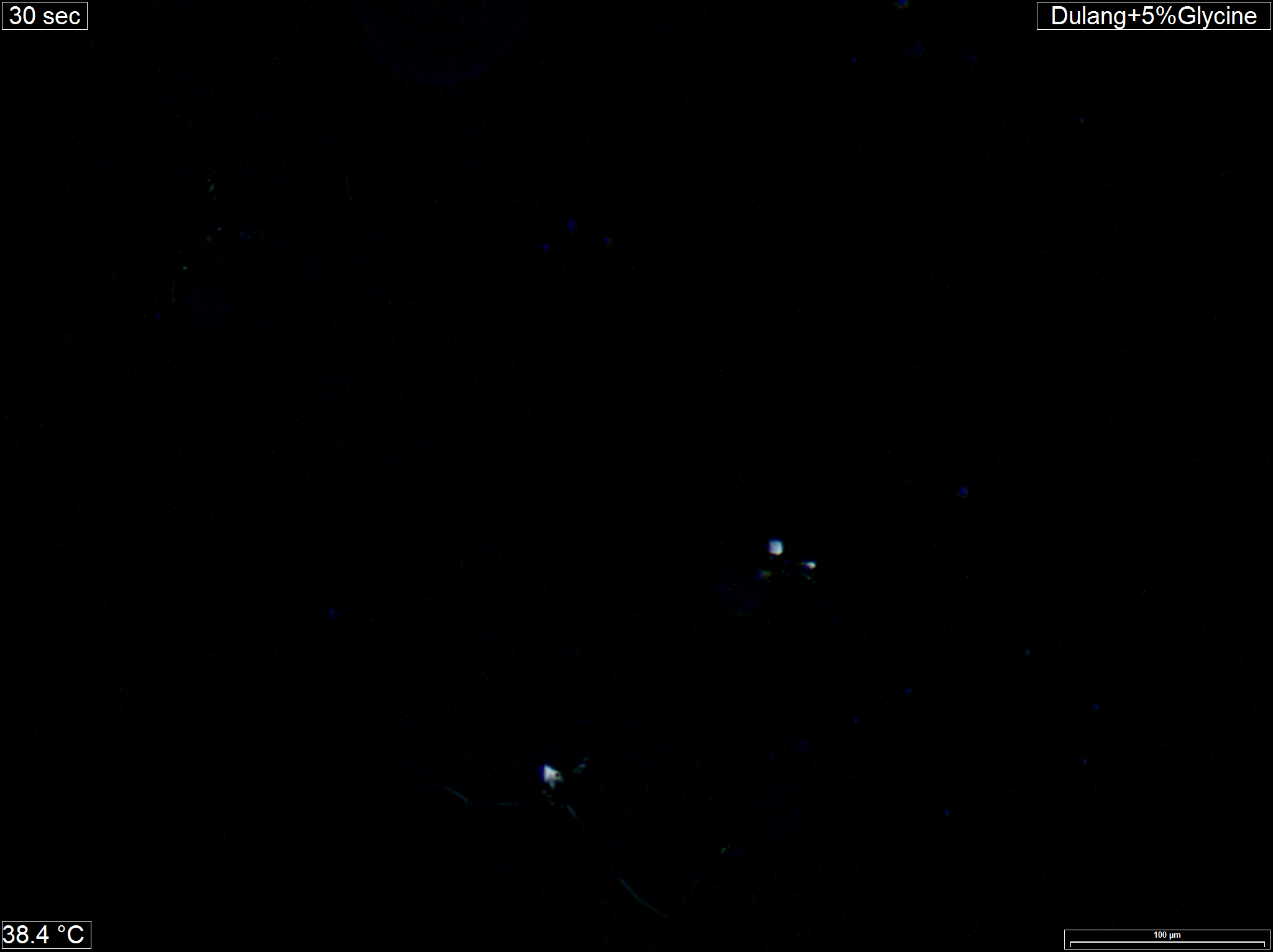

Supplement: S1 Data — (ZIP) [file pone.0313394.s001.zip › Data/CPM/Dulang+5%Glycine_002 (1).tif]

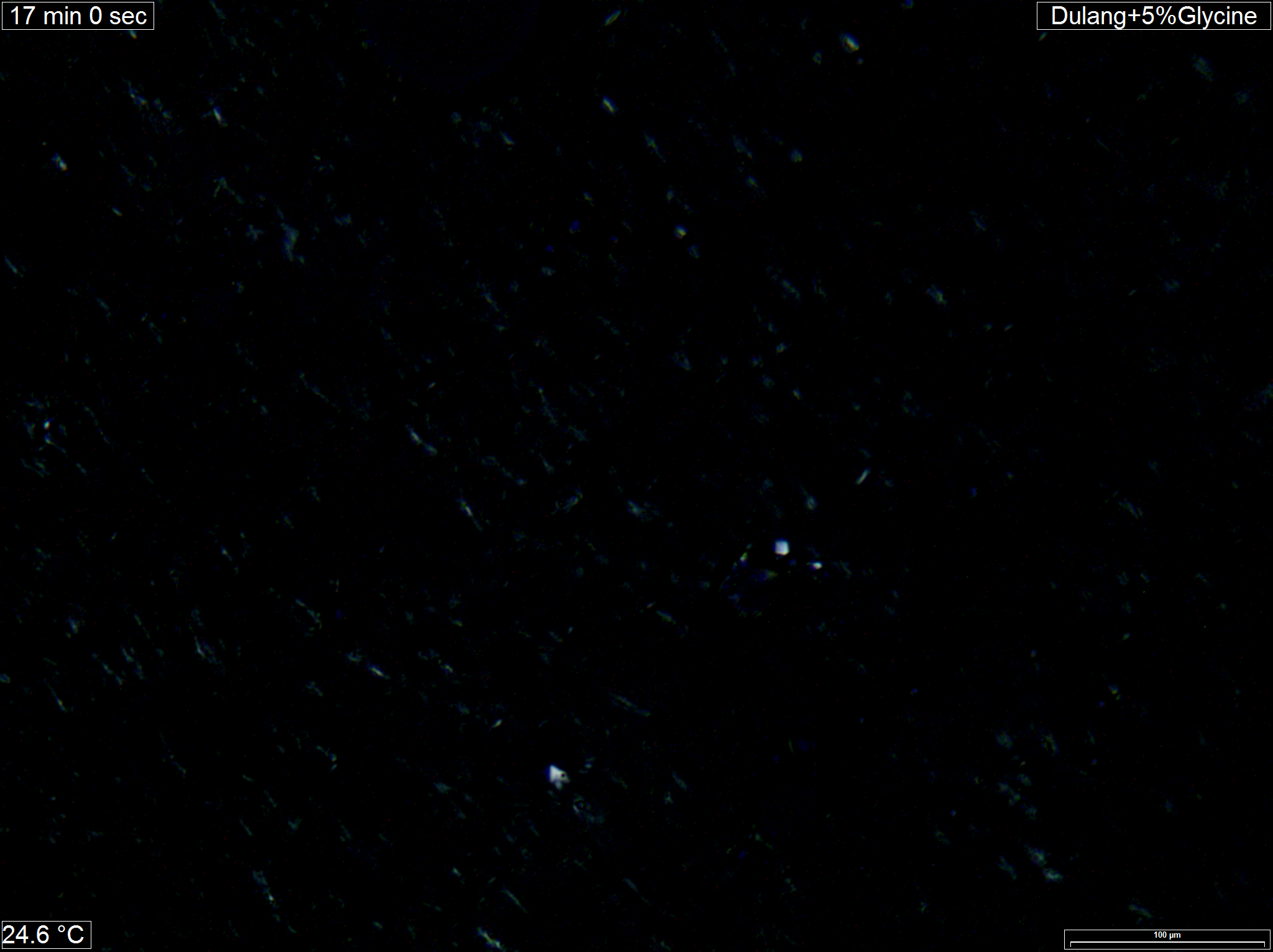

Supplement: S1 Data — (ZIP) [file pone.0313394.s001.zip › Data/CPM/Dulang+5%Glycine_035 (1).tif]

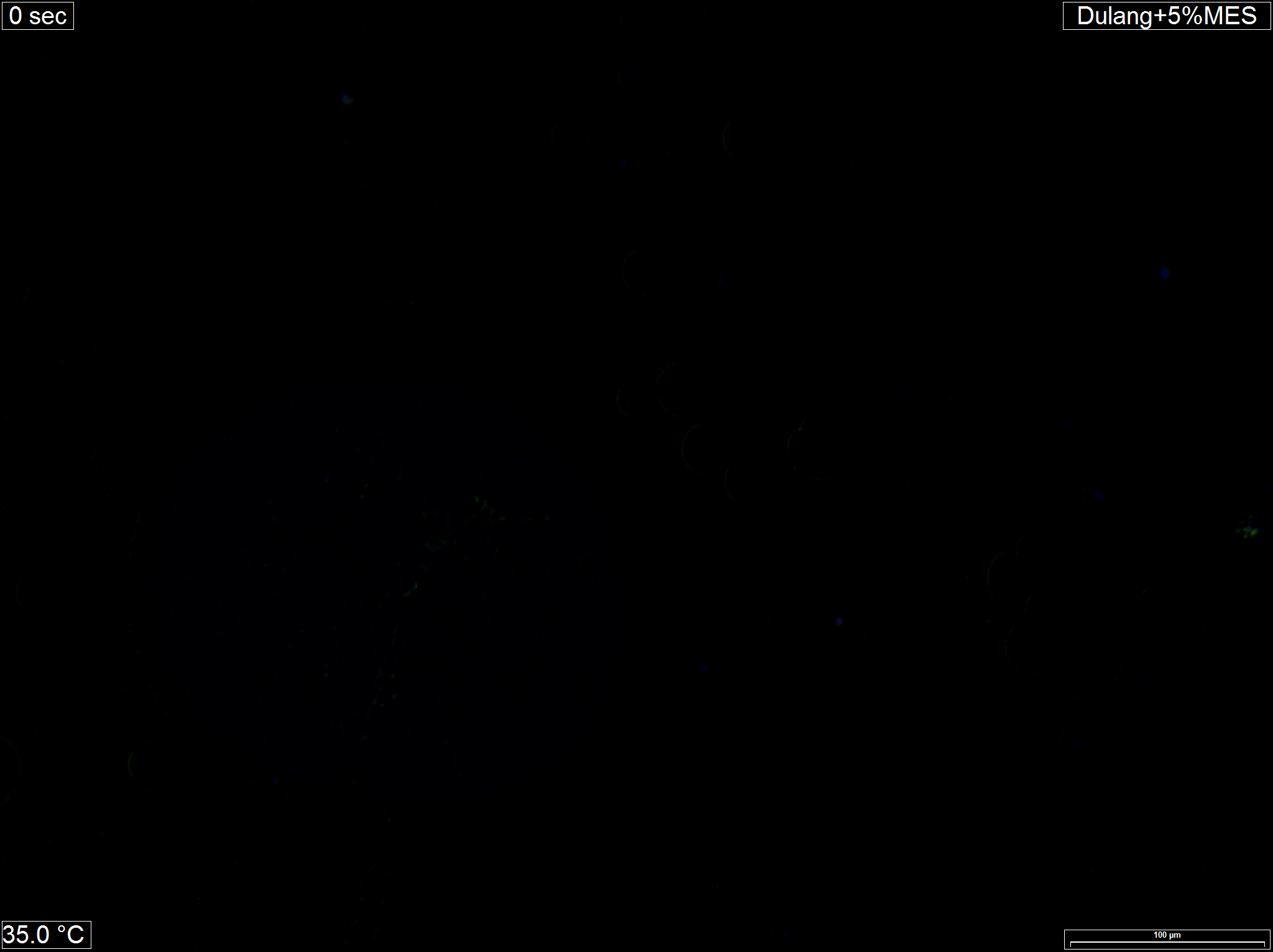

Supplement: S1 Data — (ZIP) [file pone.0313394.s001.zip › Data/CPM/Dulang+5%MES_001 (1).tif]

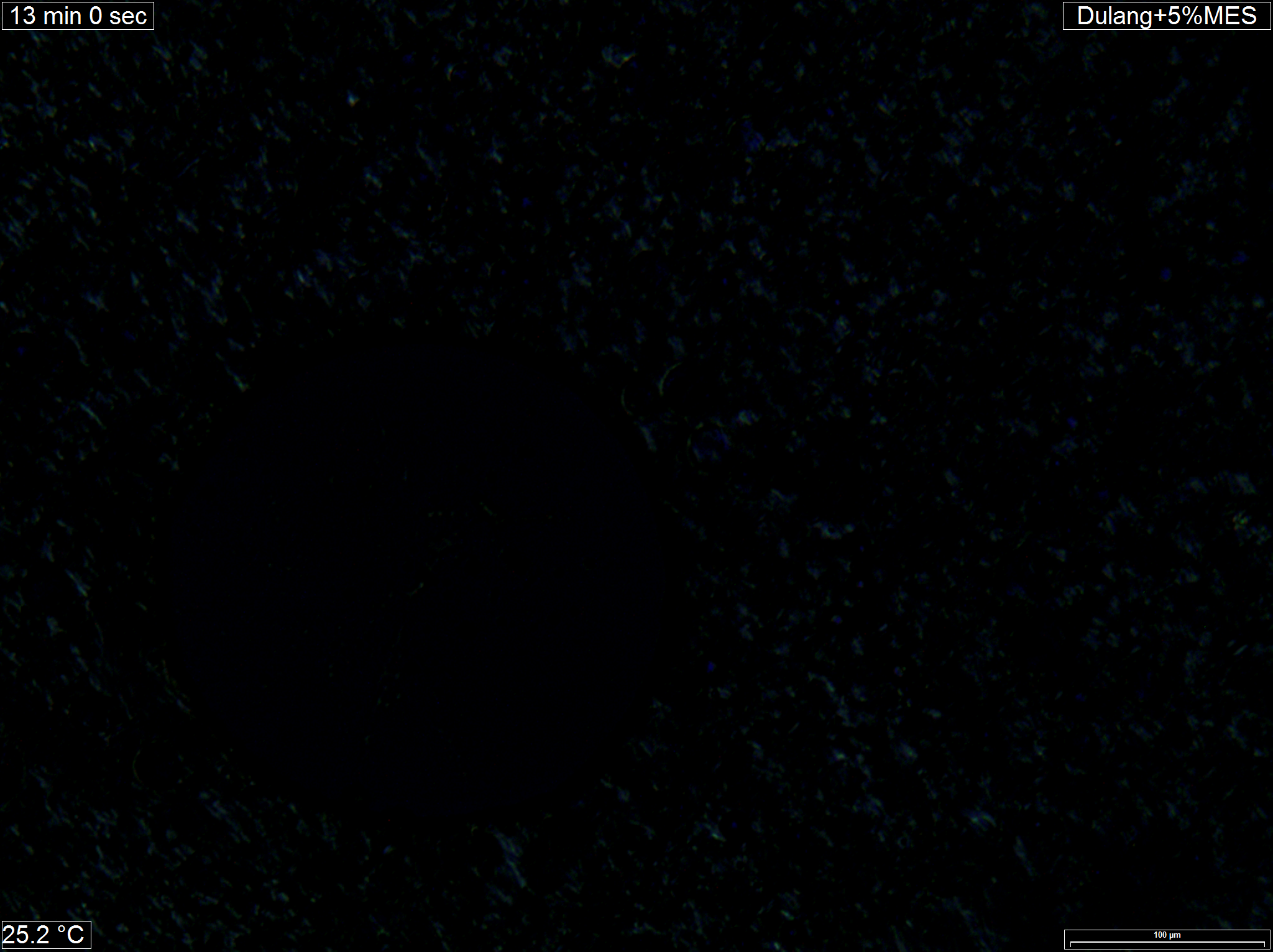

Supplement: S1 Data — (ZIP) [file pone.0313394.s001.zip › Data/CPM/Dulang+5%MES_027 (1).tif]

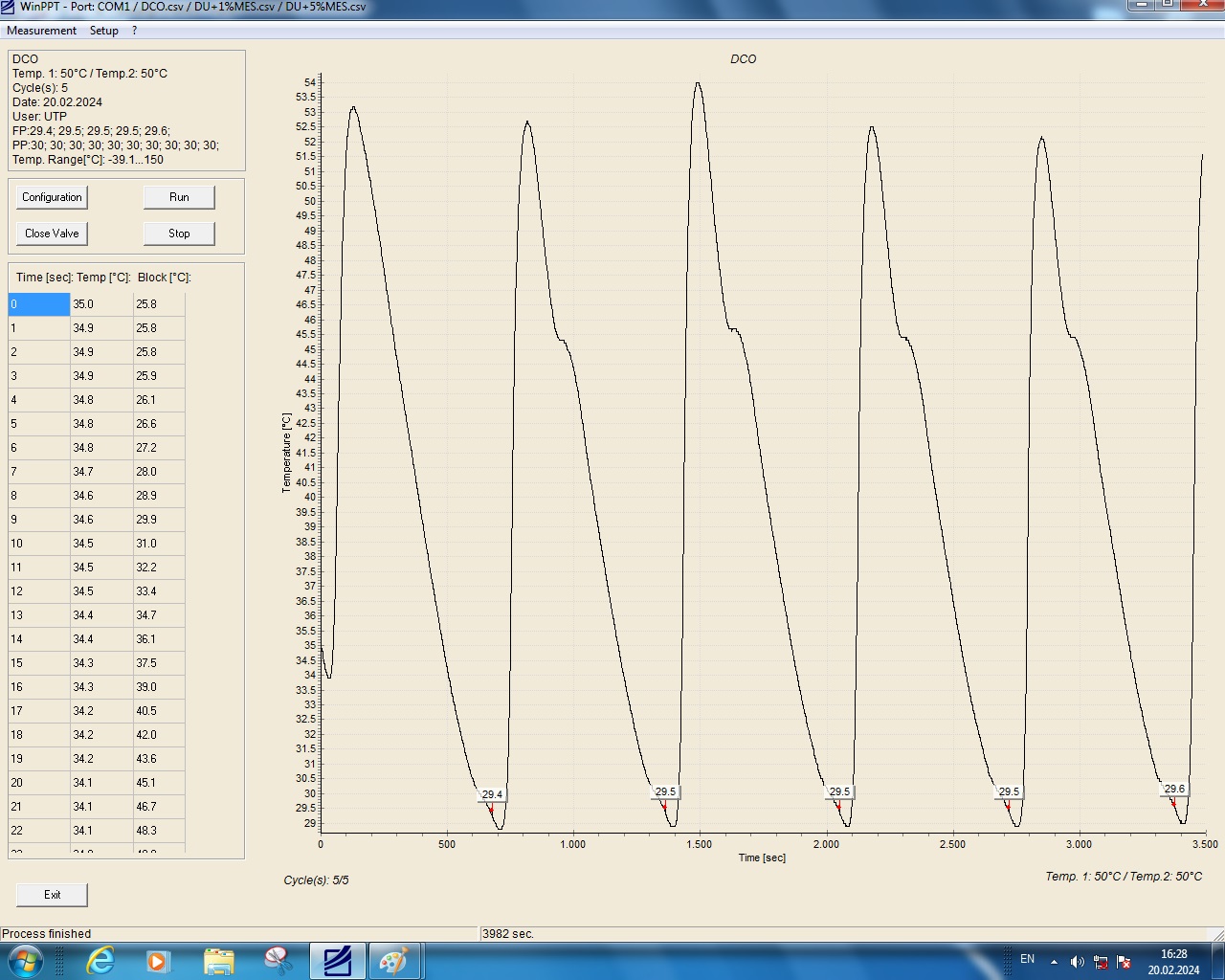

Supplement: S1 Data — (ZIP) [file pone.0313394.s001.zip › Data/PPT/DCO.jpg]

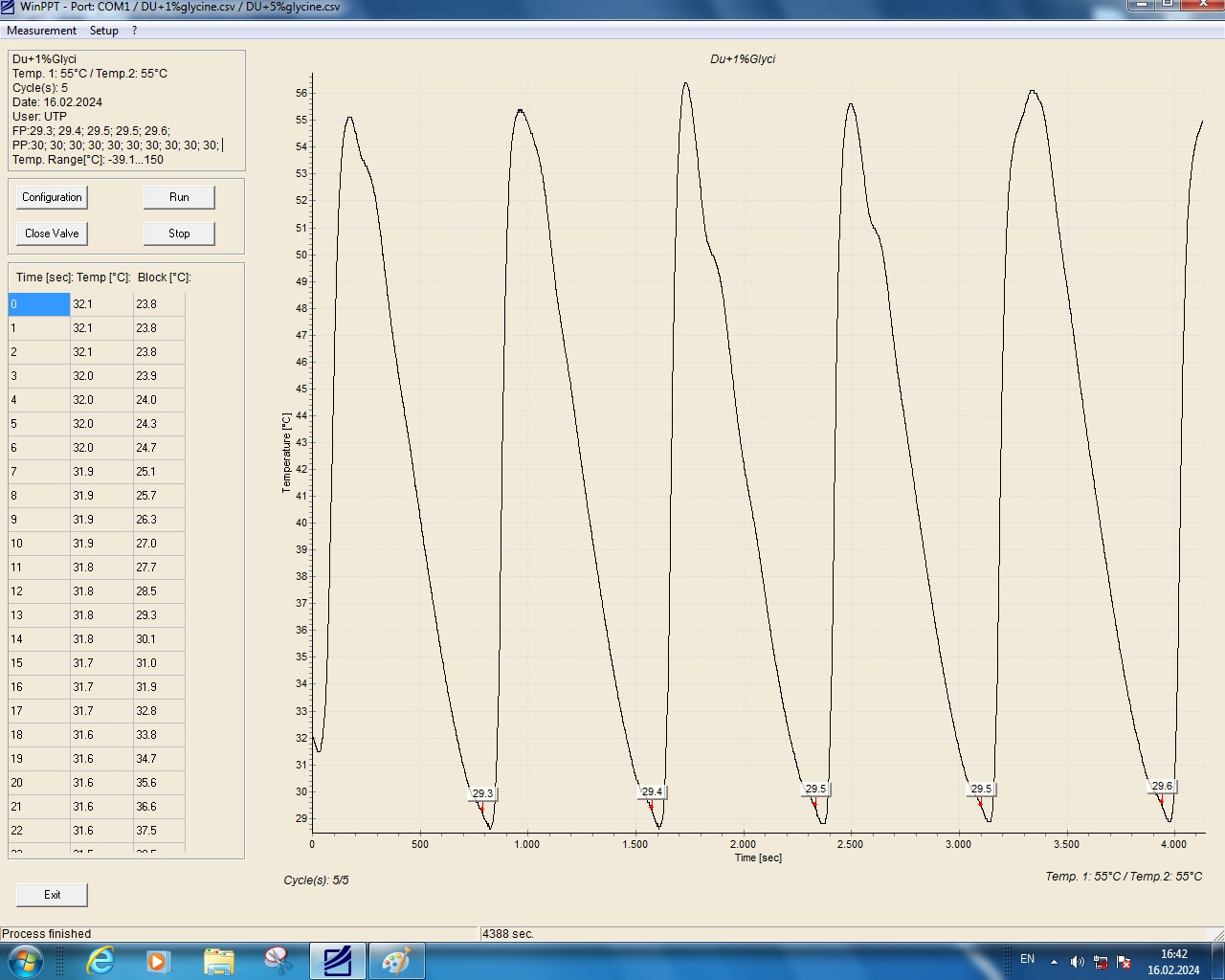

Supplement: S1 Data — (ZIP) [file pone.0313394.s001.zip › Data/PPT/Dulang+1%Glycine.jpg]

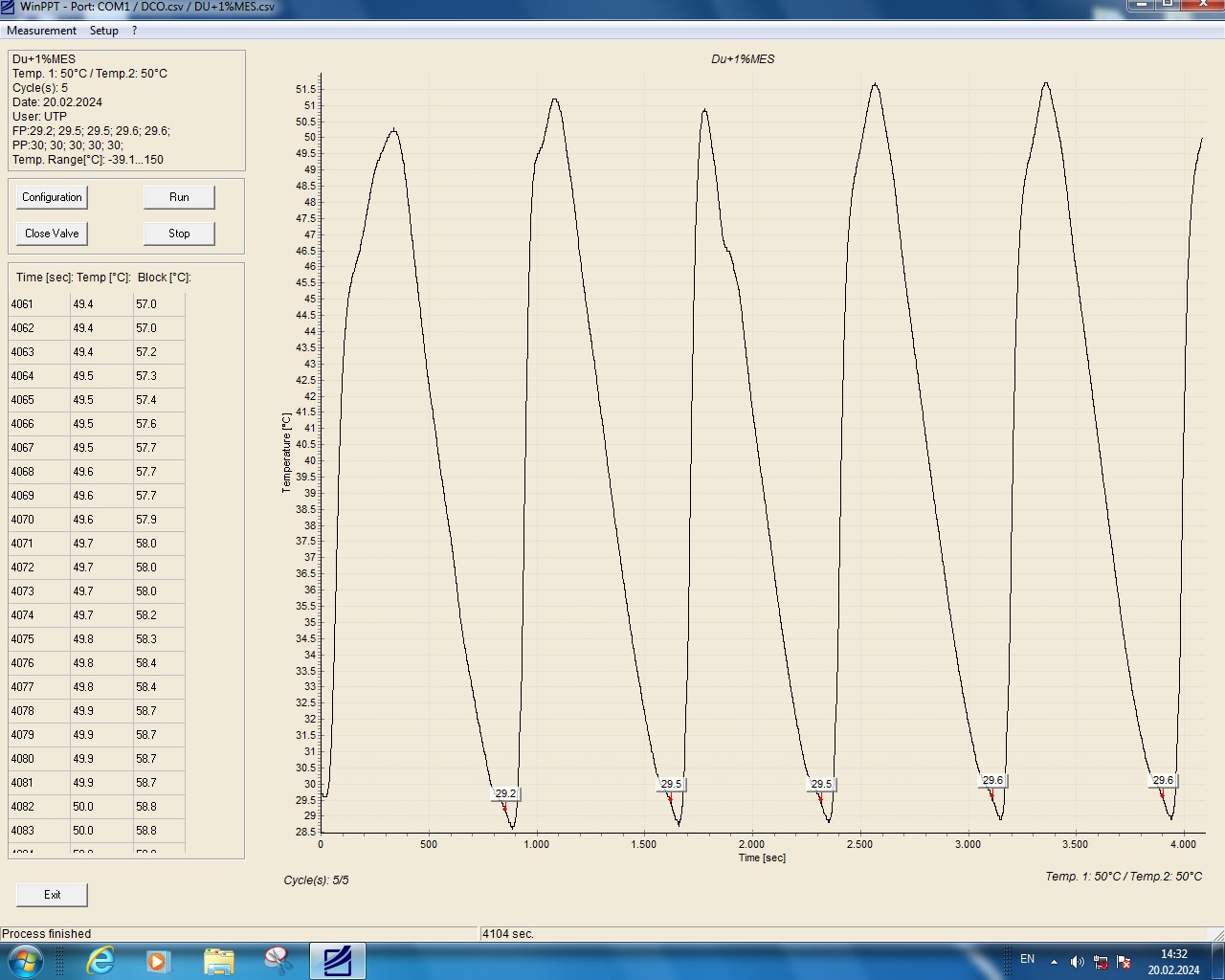

Supplement: S1 Data — (ZIP) [file pone.0313394.s001.zip › Data/PPT/Dulang+1%MES.jpg]

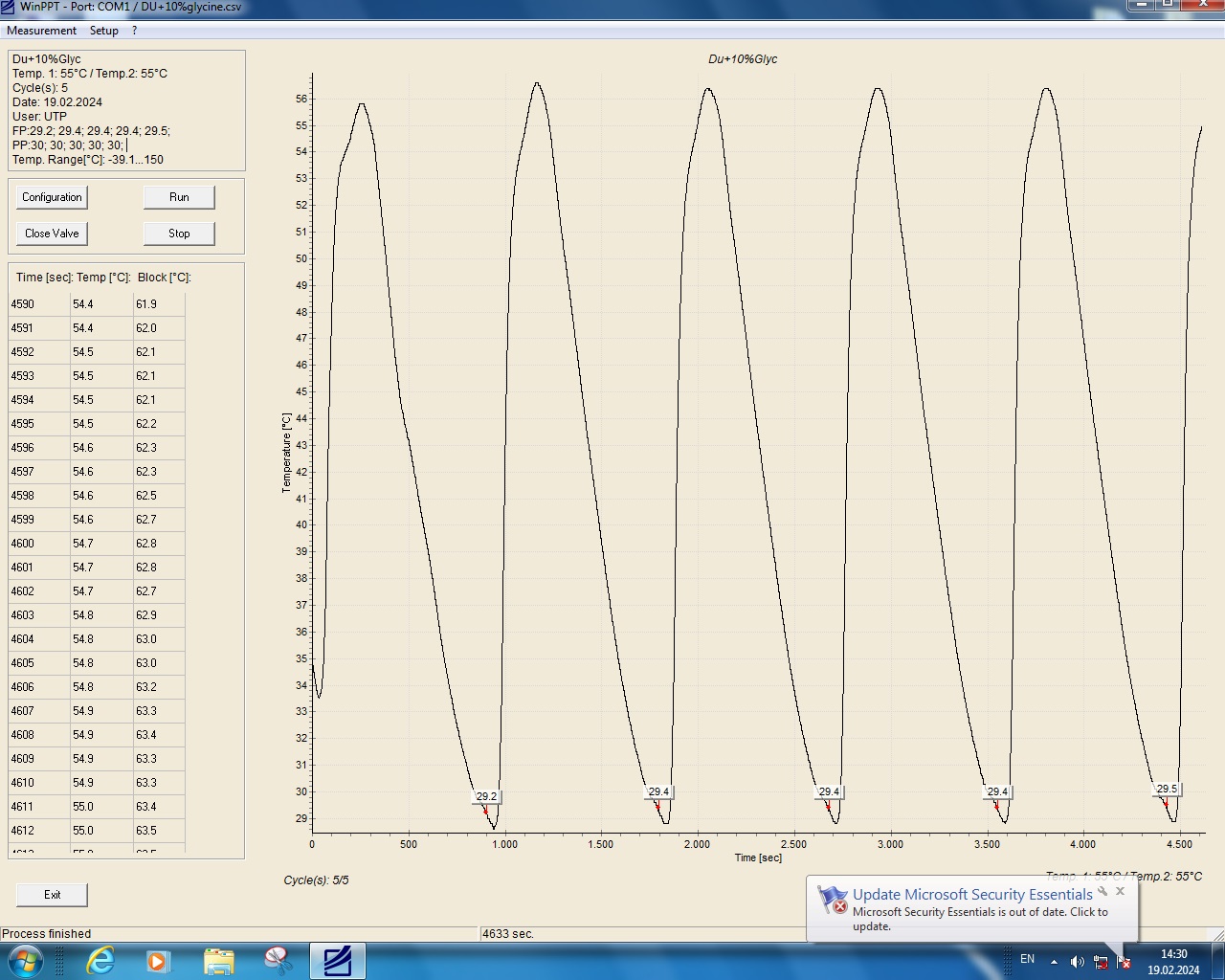

Supplement: S1 Data — (ZIP) [file pone.0313394.s001.zip › Data/PPT/Dulang+10%Glycine.jpg]

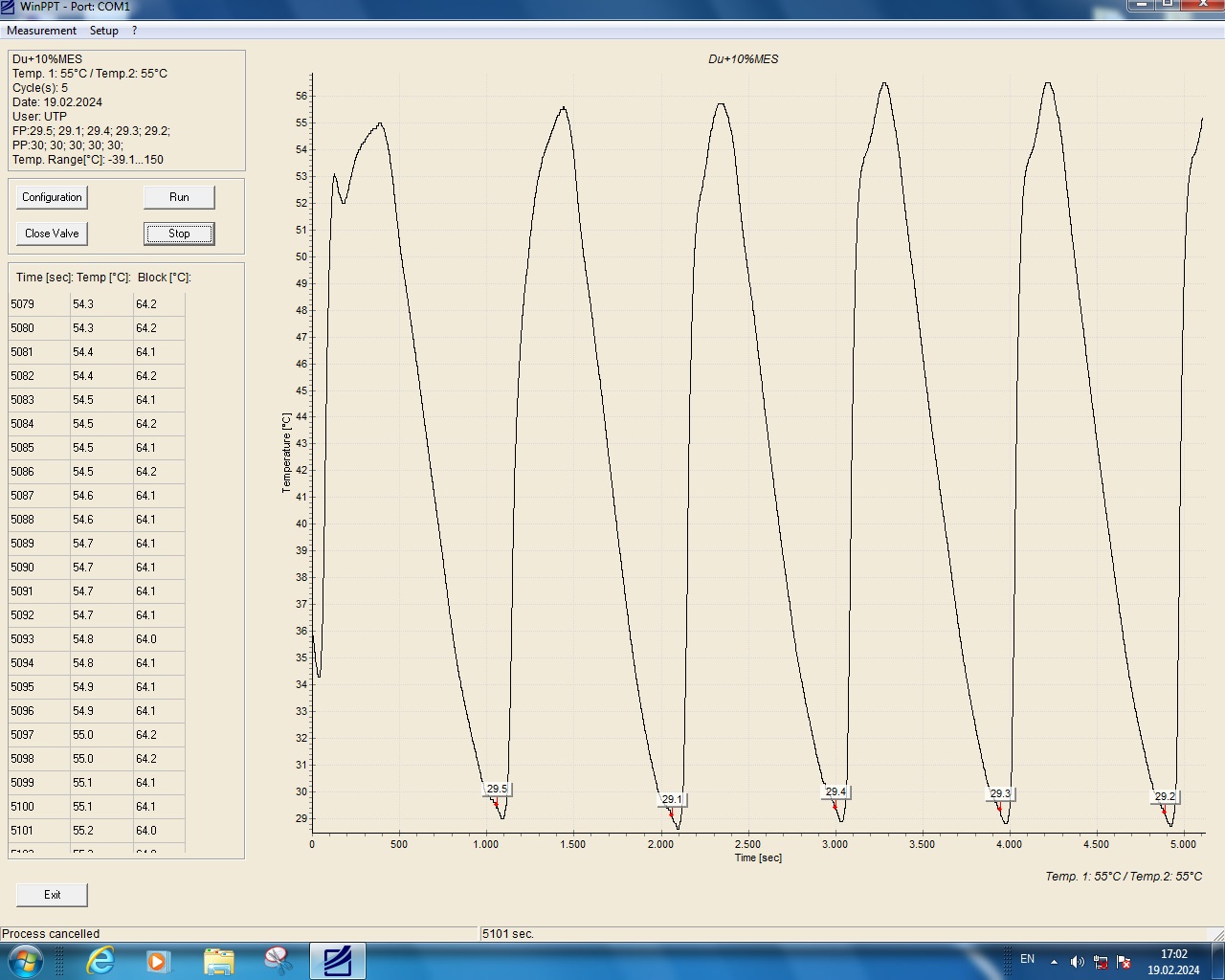

Supplement: S1 Data — (ZIP) [file pone.0313394.s001.zip › Data/PPT/Dulang+10%MES.jpg]

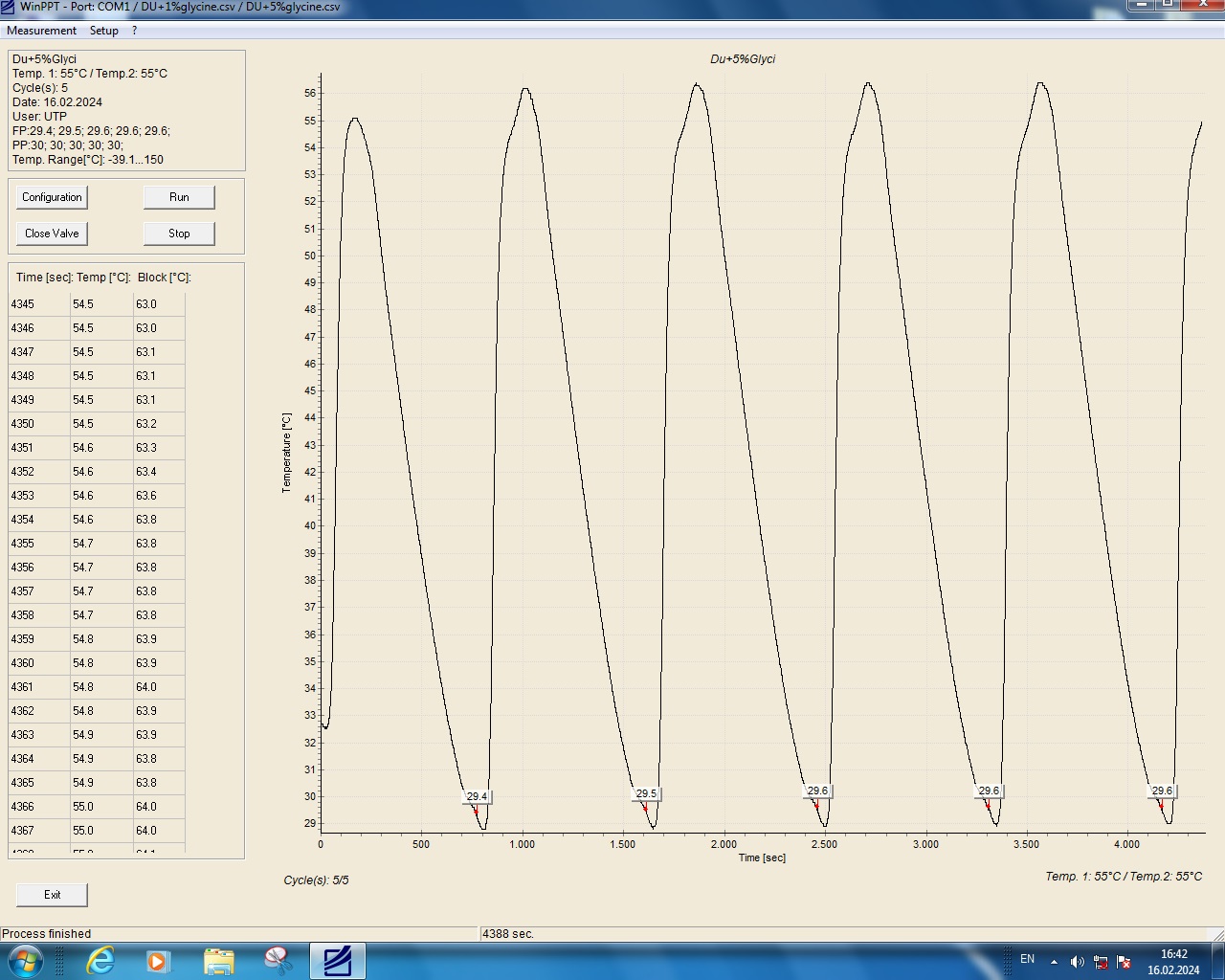

Supplement: S1 Data — (ZIP) [file pone.0313394.s001.zip › Data/PPT/Dulang+5%Glycine.jpg]

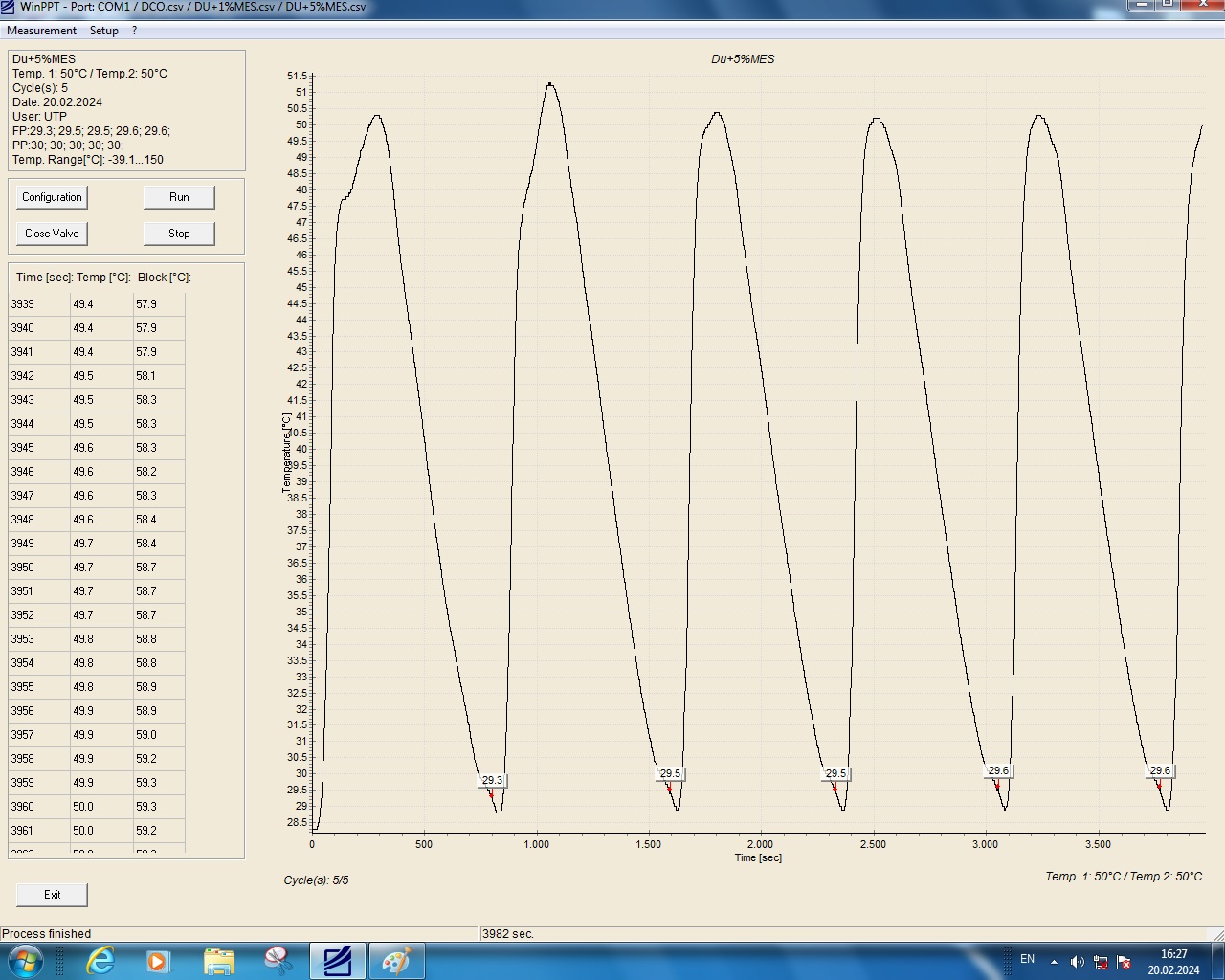

Supplement: S1 Data — (ZIP) [file pone.0313394.s001.zip › Data/PPT/Dulang+5%MES.jpg]
